# Exploring the rise and diversity of health and societal issues that use a public health approach: A scoping review and narrative synthesis

**DOI:** 10.1371/journal.pgph.0002790

**Published:** 2024-01-10

**Authors:** Alison Hurst, Nick Shaw, Daniele Carrieri, Ken Stein, Katrina Wyatt

**Affiliations:** 1 Faculty of Health and Life Sciences, Department of Health and Community Sciences, Relational Health Group, University of Exeter Medical School, Exeter, United Kingdom; 2 Gloucestershire Hospitals NHS Foundation Trust, Gloucester, United Kingdom; 3 National Institute for Health Research Applied Research Collaboration South West Peninsula (PenARC), University of Exeter Medical School, University of Exeter, Exeter, United Kingdom; Babcock University, NIGERIA

## Abstract

There is an increase in calls across diverse issues for a “public health approach” however, it is not clear whether there is any shared understanding in approach in its conceptualisation or implementation. Our aims were to (1) identify and categorise the issues which discuss a public health approach within published literature since 2010, (2) chart the descriptions and applications of public health approaches across and within four purposively sampled categories of issues, and (3) capture any evaluations conducted. A scoping review of published literature was undertaken; Seven leading databases were searched: AMED, APA PsycInfo, ASSIA, CINAHL complete, Cochrane Library (Review), Embase, and MEDLINE for articles published between 2010 and 2022 which have applied, described or called for a “public health approach” to address any issue. 3,573 studies were identified through our initial searches, of these 1,635 articles were recognised for possible inclusion from analysis of titles and abstract. The final number of included studies was 1,314. We identified 28 categories, 26 of which were societal issues, where a public health approach is being advocated. We purposively selected four of these categories; adverse childhood experiences; end of life care; gambling addiction and violence reduction/ knife crime for further analysis of the approach including how it was conceptualised and operationalised; less than 13% of the studies described the implementation of a public health approach and there was considerable heterogeneity across and within categories as to how this was done. Since 2010 there have been increasing calls for a public health approach to be taken to address health and societal challenges. However, the operationalisation of a public health approach varied extensively and there were few evaluations of the approach. This has implications for policy makers and those involved in commissioning related approaches in the future as the evidence-base is limited.

## Introduction

In 1920 Winslow defined public health (PH) as “the science and art of preventing disease, prolonging life and promoting physical health and efficiency through the organized community efforts” [1]. Hence the focus of PH is broad; from responding to urgent threats to health (such as infectious disease outbreaks) to preventing ill health, as well as seeking to create the conditions for addressing inequalities and supporting healthy populations. From its inception as a distinct discipline in Europe (EU) and United States of America (USA) in the nineteenth century, PH attempted to apply scientific understanding to the causes of diseases and acknowledged that universal measures (rather than individual treatment approaches) were needed to prevent and address the causes of infectious diseases. Hanlon and colleagues characterises the nature of PH problems and the approach PH has taken to address them as waves, with each wave reflecting both societal conditions as well as scientific understanding of the cause of the problem [2]. So, whilst the initial waves were in response to halting disease progression, identifying the causes of diseases and vaccination programmes, subsequent waves have tried to address the increase in morbidity and mortality from non-communicable diseases, focusing on the conditions in which people live and work and developing interventions to change people’s behaviours. Hanlon and colleagues highlight that the current (fifth) wave needs to respond to problems which are different in kind to the previous four, suggesting such problems are ‘wicked issues’ (issues which have complex interdependencies, where there is difficulty in defining the nature of the problem, and identifying and reaching a consensus on how to address it) [3]. These problems, such as climate change and inequalities, require PH to take a complex systems approach to understanding the nature of the problem and affecting the conditions which have given rise to it.

The timing of this so-called fifth wave of PH coincided with calls from the World Health Organisation (WHO) to recognise a broad range of harms arising from societal issues (for example violent crime, gambling related harms) as well as certain aspects of health care (for example end of life care) as PH problems, and to take a PH approach to addressing them [4–6]. The pillars of the WHO PH approach include: defining the magnitude of the problem, identifying the risk and protective factors, and applying prevention strategies, whilst considering widespread adoption [7]. Hence a PH approach recognises the scale as well as the complex nature of the problem and the need for cross-sector working and community engagement to understand the nature of the problem and address the underlying determinants.

Probably the most well-known example of an issue that is being addressed using a PH approach is that of preventing and addressing violence. This interest originated in the USA where the scale and magnitude of the problem led it to be included as a priority area for action in the Surgeon General’s Report, Healthy People (1979) [8]. The goals for violence prevention detailed in this report were translated into measurable objectives in Promoting Health/Preventing Disease: Objectives for the Nation [9]. Over the next couple of decades the PH approach to violence shifted from describing the problem to understanding what worked in preventing it, and by 1993, a plethora of violence-prevention programmes had been developed and undertaken in schools and communities across the USA. This work led to the creation of the Scottish Violence Reduction Unit (VRU) in 2005, and of the English VRUs in 2019. A central tenet of the approach is the “cooperation and integration across public health, health care, mental health, criminal justice, social service, education, and other relevant sectors” to respond to the problem [10]. Since then this collaborative approach has also been advocated for a plethora of societal issues including; modern day slavery, child abuse and neglect, gambling addiction and EoL care [11,12].

Whilst it is clear that an increasing number of issues are calling for a PH approach to be taken, it is not clear whether there is a shared understanding of what the term means or how it is operationalised or evaluated. Given the focus on system approaches to understanding and addressing population health issues and the apparent increase in calls for a PH approach to address these issues, we sought to undertake a scoping review and narrative synthesis to: (1) systematically search the scientific literature to identify and categorise the issues where a PH approach is being described, (2) chart the nature of the descriptions and applications of PH approaches across and within four purposively sampled categories of issues over the last decade, and (3) capture any evaluations of the PH approach as well as any descriptions as to how it can be scaled up.

## Methods

### Design

We selected a scoping review methodology for our approach as we wanted to examine the range and nature of the use of the term “public health approach” and its application.

### Search strategy

Our inclusion criteria were very broad: We looked for the term “public health approach” within the title or abstract of any published research. The publication date was set to examine the last decade limited to January 2010 to September 2021 which was then updated to June 2022. This pragmatic time period was chosen to provide a snapshot of the developing field of inquiry that referred to a PH approach. The databases searched were Allied and Complementary Medicine (AMED), American Psychological Association’s PsycInfo, Applied Social Sciences Index & Abstracts (ASSIA), the Cumulative Index to Nursing and Allied Health Literature (CINAHL) complete, Cochrane Library (Review), Excerpta Medica Database (Embase), and the US National Library of Medicine’s biomedicine bibliographic database (MEDLINE). Results were uploaded to an Endnote library.

### Study selection

We applied minimal restrictions to included studies in order to be as inclusive as possible. We excluded conference, posters and meeting abstracts, book reviews and any entries such as errata or introductions to other studies. Studies were also excluded if they related to training of, or the curriculum for, health or other professionals. Dissertation abstracts were also excluded if their main area of interest was already highlighted in other studies. The abstracts needed full text in English to be included in the sampled categories for the additional charting.

### Data charting

Included study details were recorded on a Microsoft Excel spreadsheet capturing; Authors, Title, Journal, Year, geographical location, article type and area requiring a PH approach. Where possible we grouped issues into wider categories of the nature of the issue under investigation. We purposively sampled four of these categories to reflect four distinct societal issues for further exploration. We extracted additional details from these categories including: the study aims, the rationale behind the PH approach, whether focus was on primary, secondary or tertiary prevention, the anticipated impact and whether any evaluation was carried out and if they attempted to deliver it at scale.

### Data synthesis

For each included study, the primary area of interest was recorded and categorised according to the overall subject area. These higher-level categories were discussed by two authors and subject areas that were covered by only a small number of studies (three or less) were added to an ‘other’ category to be discussed separately. Of the studies whose focus seem to cover more than one high level category, for example mental health and suicide, the study abstract was reviewed and an agreement as to the main subject was reached between the same two authors.

Similarly, there was cross-over in subject areas with zoonotic diseases which are both infectious/ communicable diseases as well as environmental health hazards. For the purpose of this review and to maintain consistency, we have used the former category. We considered adverse childhood experiences to include child abuse and neglect as this category was related to the age of population, whereas slavery and exploitation can occur at any time over the life course.

We purposefully sampled four subject categories (Adverse Childhood Experiences (ACEs), End of Life (EoL) care, gambling harms, and violence including crime and delinquency) for further analysis. We classified each study according to whether they made a call for PH approach or reported aspects that a PH approach needed to consider or provided a description or an application of the PH approach. The title and abstract were initially screened by the lead author with some full texts being retrieved where the abstracts were unavailable or not present. The other authors were secondary reviewers to check for consistency, and any disagreements were discussed, clarified and resolved.

Reviews were searched for potential studies that may involve an application or operationalisation of a PH approach for the four subject areas, the title and abstract of their included studies were searched for additional studies or reports that fulfilled our inclusion criteria. In addition, where a study seemed to refer to a single specific report, the report was sought and included.

A narrative synthesis approach was used to identify the framing and features from the included implementation studies across the four subject areas. A narrative synthesis uses a textual rather than a statistical approach for analysing results and drawing conclusions. This words-based approach is more suitable to exploring the rationale behind taking and PH approach and its operationalisation. In particular we looked at whether the operationalisation of a PH approach differed in subject areas where the call for such an approach was more recent, compared with areas such as preventing violence where a PH approach has been in place for several decades, thus hypothesising that the more established areas would include more operational approaches.

## Results

**Fig 1:**
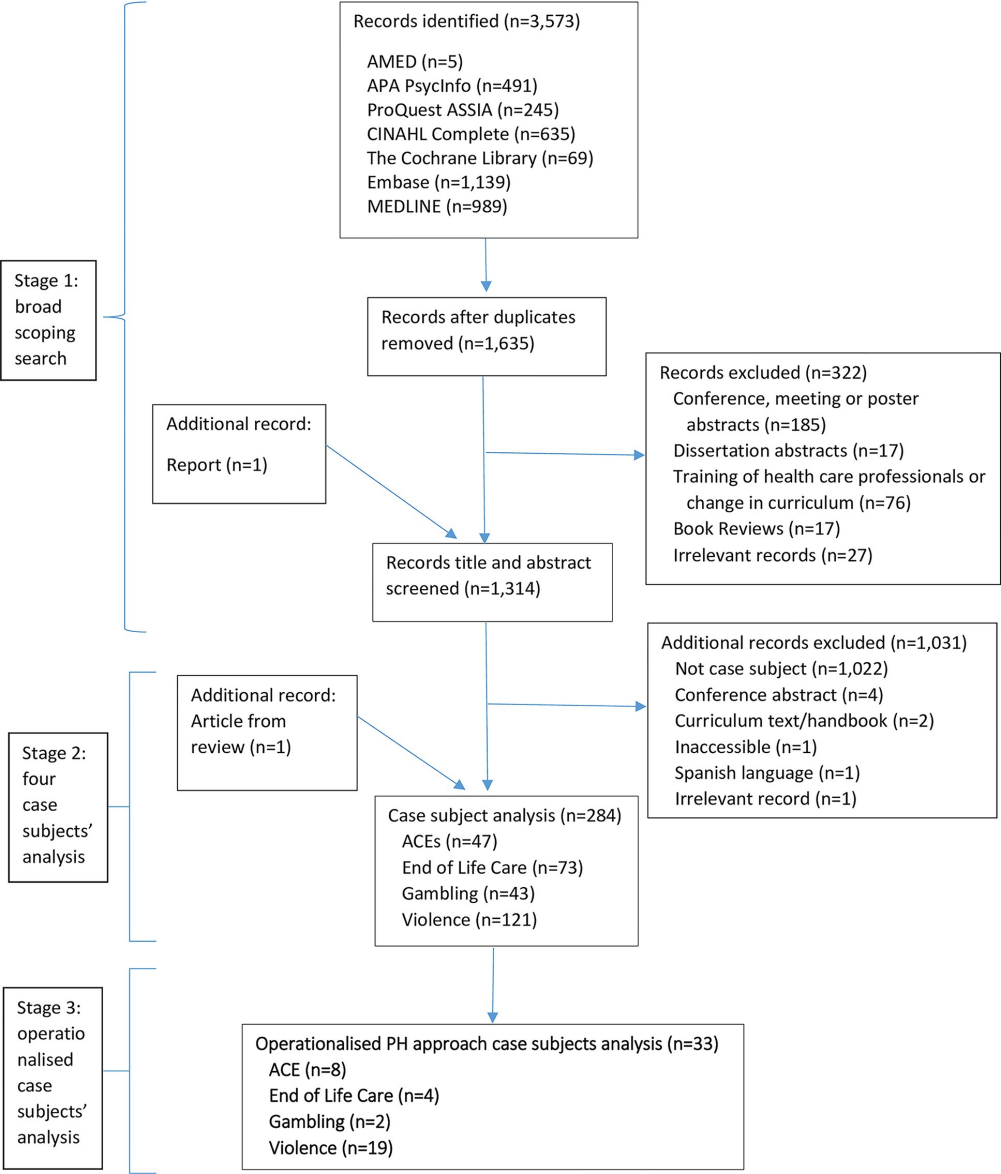
Flow diagram that summarises our search strategies.

One thousand three hundred and fourteen studies were included in this review, see Fig 1.

Studies were grouped into categories according to their primary focus, see Table 1.

**Table 1:** The number of included studies by subject category with the geographical locations of the studies.

Subject Category	No. of studies	Geographical locations of studies
Eye care	7	USA, Saudi Arabia, South Africa, UK, India
Health literacy	7	USA, Turkey, Hong Kong
Slavery and exploitation	9	USA, India, UK, Canada
Additional needs/ support	10	Pacific Islands, UK, USA
Workplace health	10	Macedonia, USA, France, Italy, UK
Terrorism and terrorist attacks	11	USA, UK, Nebraska
Oral health	12	UK, Turkey, USA, Australia, Italy, France
Chronic disease and pain	13	USA, Australia, New Zealand, Europe, Sub-Saharan Africa
Physical activity	13	Montenegro, Brazil, UK, USA, Germany, LMICs, Scandinavia
Disaster response	14	India, USA, Japan, England
Weight management	16	Poland, Australia, USA, Mexico
Dietary health	21	UK, Germany, USA, Ireland, India, Canton Sarajevo, Thailand, Mozambique, Germany, New Zealand, (Hungary, Poland, and Ukraine), England, Japan, Kenya
Environmental health	23	EMR, Brazil, Italy, Canada, USA, Arkansas, India, South Africa, Portugal, Columbia, China, Nigeria, Japan
Ageing	28	Mexico, UK, USA, Canada, Japan, Ghana, India
Population health	39	Britain, USA, India, UK, Canada, France, Europe, People’s Republic of China and Eastern Asia, West Africa
Injury prevention	40	Saskatchewan, China, Canada, Qatar, Middle Eastern country, USA, Hong Kong, UK, Australia, Iran, American Indian, Ethiopia, Lithuania, Kathmandu Nepal, Alaska
Gambling	43	Canada, New Zealand, Wales, Switzerland, India, Spain, Australia, UK, Finland, USA
Reproductive and sexual health	43	USA, Kenya, Canada, Ireland, Australia, India, Japan, South Africa, Arab League and diaspora, Serbia
Adverse Childhood Experiences	47	Low and middle income countries (LMICs), USA, England, Australia, Canada, UK, Singapore, Netherlands, Europe
COVID-19 (Kept separate due to newness of issue)	48	High income countries (HICs), Italy, Sweden, USA, China, Democratic Republic of Congo, Canada, England, India, Africa, UK, Australia
Other*	50	Egypt, UK, USA, Saudi Arabia, South Africa, Australia, South Africa, Turkey, Malawi, Uganda, South Australia, Netherlands, Canada, LMICs, Hawaii, Germany, Belgium
Suicide prevention	55	Ghana, USA, India, Hong Kong, Canada, New Zealand
Child and young persons’ health	71	USA, China, Canada, Bangladesh, Australia, South Africa, Germany, Greece, Brazil, Argentina, Norway, Japan, Fiji and Sweden
End of Life Care	82	Brazil, Sub-Saharan Africa, UK, Australia, USA, Scotland, Portugal, Hong Kong, New Zealand, Spain, Africa, Ireland, Colombia, Canada, LMIC, Wales, India, Europe, Portugal, Argentina, Zimbabwe, Britain, South Asia
Mental health	84	Mozambique, Brazil, Mexico, North Africa, USA, India, Cuba, Netherlands, Australia, South Africa, UK, Chile, Germany, Hong Kong, Europe, New Zealand, Australia, LMICs, Columbia, Middle East, Europe
Non-communicable diseases	87	Canada, Europe, Iran, India, USA, Sweden, UK, Mongolia, Africa, South India, Uganda, South-East Asia, Ghana, Italy, France, Mexico, Kuwaiti, India, Nigeria, Australia, Denmark, Malaysia, India, Nigeria, Brazil, Nepal, China
Violence	120	USA, Spain, Chile, UK, Latin America, Afghanistan, Qatar, Philippine, South Africa, Iraq, Germany, Australia, Romania, Scotland
Substance abuse	142	Sub-Saharan Africa, Ireland, Thailand, Asia, Canada, USA, UK, South Africa, France, Nordic Countries, Greenland, India, North America, Colombia, Mexico, Australia, New Zealand, Italy, Thailand, Finland, Asia, Botswana, Sri Lanka, Alaska, North America, Portugal, Iran, Alaska, Kenya, Malaysia.
Communicable diseases	169	Israel, Australia, Brazil, LMIC, USA, India, Cameroon, Africa & Asia, Japan, Zimbabwe, Madagascar, Ghana, West Africa & USA, China, Portugal, Togo Congo, Uganda, Americas, Europe, Russia, China, S. America and Asia, Uganda, Ethiopia, West Africa, Africa, Iceland, Spain, Malawi, Rwanda, Kenya, Sweden, Indonesia, Zimbabwe, Sub-Saharan Africa, Malaysia, England, Canada, Rhode Island, Abidjan Côte d’Ivoire, Thailand, 6 countries (China, Vietnam, Malaysia, Russia, Ukraine and USA), France, People’s Republic of China and Eastern Asia, South Africa, Eastern Europe.

* The ‘other’ category includes: allergy prevention, antimicrobial resistance, clubfoot, cognitive enhancement, correctional health care, drug adherence and management, family courts/ divorce, food poisoning/ food safety, gender and sexual dissidents in regards to political disengagement, genomics, health data research, hearing impairment, herbal drugs, homelessness, illegal wildlife trade/ biodiversity loss, immigrants and asylum seekers health, internet addiction, loneliness (all ages), mass incarceration of pregnant & parenting women, musicians wellbeing, prescription drug adherence, prisoner re-entry, rare diseases, school readiness, sleep duration, social care, socially responsive physiotherapists, stalking, sudden cardiac arrest, surgical care, transgender immigrants, universal health coverage.

Table 1 details the number of studies per subject category with the geographic locations of the studies. (Total n = 1314) In total we had 28 subject categories, 26 were societal issues, two were related to health and communicable diseases and we created an additional group named ‘other’. The most common categories were communicable diseases (13%), substance abuse (11%) and violence (9%). Mental health, non-communicable diseases and EoL care were the next most common subjects having 84, 86, 82 studies respectively. Some of the ‘other’ issues could be grouped within broader categories, for example infection control for illegal wildlife trade or personalised medicine for genomics, however we kept them separate to show the extent and breadth of issues.

### Headline from Table 1: The extensive range of health and societal subject areas and broad geographical approach that advocate a PH approach

We then explored the different ways a PH approach was being discussed (e.g., aspects to consider when applying a PH approach, description of the approach or its application) for four of the categories, ACEs, EoL care, gambling related harms and violence prevention. We sought to understand whether the nature of the discussion for a PH approach had changed over the time period being examined, for each category.

Table 2 charts the studies’ focus across three time periods (2010–2013), (2014–2017) and (2018–2022).

**Table 2:** The number of studies by subject categories according to publication date and whether they make a call for, report aspects to consider, give a description or detail an application of a PH approach.

	Category	(2010–2013)	(2014–2017)	(2018–2022)
**Adverse childhood experiences (ACEs) (n = 47)**	*Number of studies*	*16*	*17*	*14*
	calling for a PH approach	3 (19%)	3 (18%)	0
	Highlighting aspects to consider	6 (38%)	8 (47%)	7 (50%)
	Description of the approach	5 (31%)	3 (18%)	4 (29%)
	Implementation of the approach	2 (13%)	3 (18%)	3 (21%)
**End of life (EoL) care (n = 73)**	*Number of studies*	*14*	*21*	*38*
	calling for a PH approach	1 (7%)	1 (5%)	5 (13%)
	Highlighting aspects to consider*	10 (71%)	12 (57%)	24 (63%)
	Description of the approach	2 (14%)	8 (38%)	6 (16%)
	Implementation of the approach	1 (7%)	0	3 (8%)
**Gambling (n = 43)**	*Number of studies*	*6*	*11*	*26*
	calling for a PH approach	1 (17%)	2 (18%)	4 (15%)
	Highlighting aspects to consider	3 (50%)	7 (64%)	13 (50%)
	Description of the approach	1 (17%)	2 (18%)	8 (31%)
	Implementation of the approach	1 (17%)	0	1 (4%)
**Violence (n = 121)**	*Number of studies*	*25*	*39*	*57*
	Calling for a PH approach	1 (4%)	5 (13%)	13 (23%)
	Highlighting aspects to consider	12 (48%)	19 (49%)	24 (42%)
	Description of the approach	7 (28%)	9 (23%)	12 (21%)
	Implementation of the approach	5 (20%)	6 (15%)	8 (14%)

*It should be noted that two papers in this group provided some limited evaluation of end-of-life care service providers about the uptake of PH approach to palliative care in the UK and New Zealand, however the focus was on barriers to implementation with inadequate details about its application.

### Headlines from Table 2: *Across the four categories there was no discernible pattern of studies advocating*, *describing or implementing a PH approach over time; Of the four subject areas*, *less than 13% of studies reported on how a PH approach was operationalised*

For all four broad subject areas, ACEs, EoL care, gambling and violence, there were relatively few studies that reported the application of a PH approach. The most common type of study (61%) suggested specific ‘aspects to consider’ with respect to what a PH approach should include. These varied within and across the four subjects, some were general and relatable to the whole population (e.g., multi-agency collaboration, proactive communication to engage media, policy makers and the public, surveillance and linkage of data systems, shift narrative while taking into account culture) whilst others were specific to high-risk populations. For example, aspects a PH approach included in violence prevention studies ranged from reducing risk factors for people considered at risk of violent behaviour, improving surveillance of places where violence was likely to occur, educating young people within schools in high-risk areas, tackling barriers and cultural contexts, incorporating the voice of the victim into management programmes.

The numbers of published studies on PH approaches for EoL care, gambling harms or violence prevention have all increased over the time period studied, see Table 2. Also, for ACEs, EoL care and violence there has been a rise in studies that reported an application of a PH approach suggesting an increased recognition of the role such approaches can play. As there were only two studies related to a PH approach to gambling it is too early to identify any trends.

**Fig 2:**
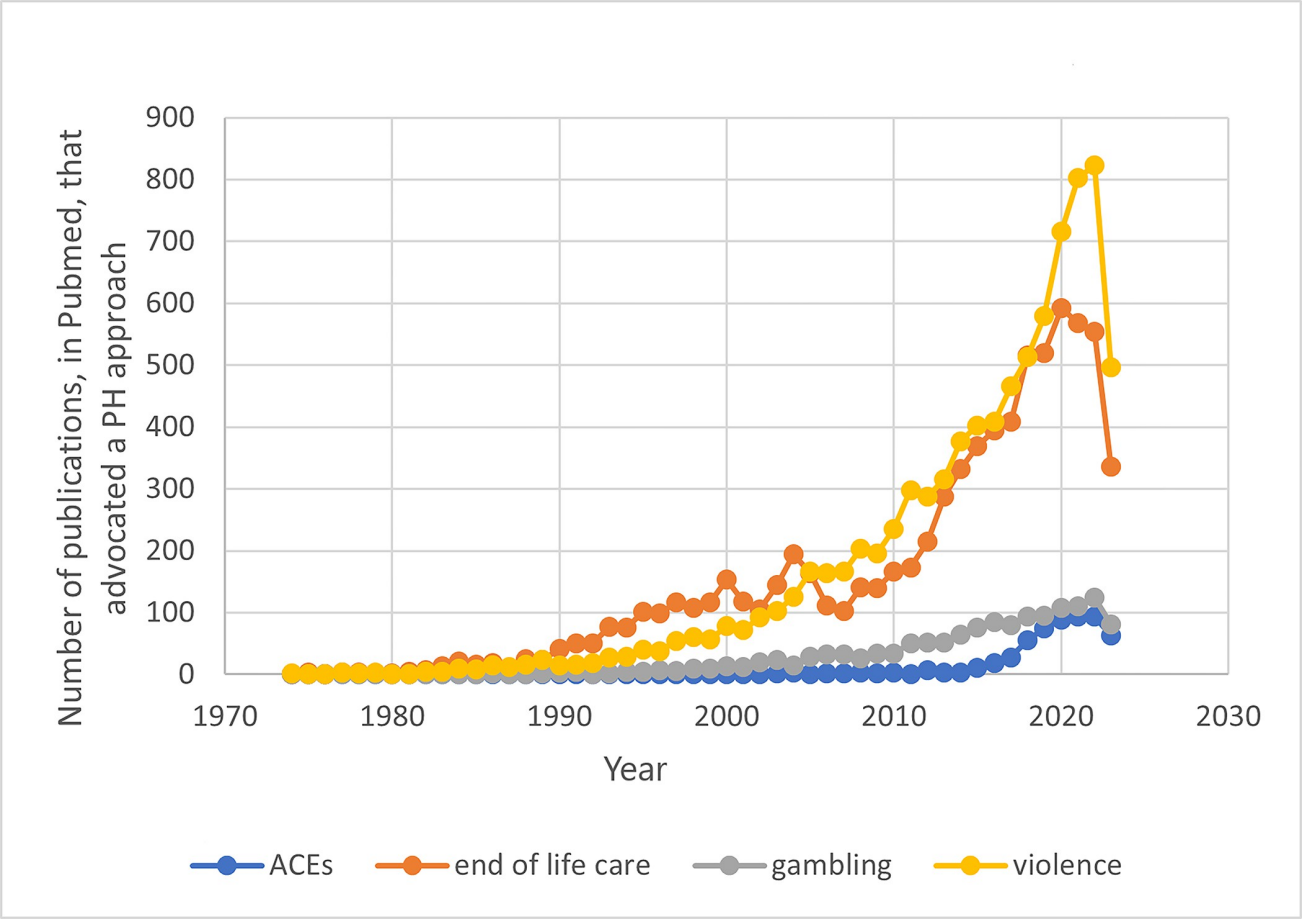
Timeline of the number of publications by area of interest.

We hypothesised that in the areas where a PH approach was more established, there would be more operationalised programmes. Fig 2 provides a snapshot from PubMed of any increase in the volume of studies discussing a PH approach for each of the four areas, showing violence and EoL care to be more established than ACEs and gambling.

For each of our four subjects of focus, we further analysed the studies that reported on the implementation of a PH approach to ascertain the rationale given for taking a PH approach as well as any details of how each approach was operationalised and any evaluations undertaken. See S1–S4 Tables for individual study details and Boxes 1–4 for key points of PH approach implementation for ACEs, EoL care, gambling and violence reduction respectively.

**Box 1. Key points from ACEs studies implementing a PH approach**

- Most PH approaches used a collection of universal and targeted approaches to preventing ACEs.
- There is wide variation in the scope and span of PH approaches, for example across 5 American states compared to 4 paediatric clinics.
- The same named approach was applied differently in different countries.
- The application of an intervention described as evidence-based often resulted in reduced evaluation even when implemented in different contexts.
- Upscaling the application of some PH approaches includes examining how transferable the previously collected knowledge and resources are to the new setting and context.

**Box 2. Key points from EoL care studies**

- The importance of collaborations and cross-sector partnerships focusing on either health care practitioners or community members acting as facilitators between the patient requiring EoL care and their family and health care services.
- The lack of evaluation results reported.
- The approaches differ in the range of services that are on offer, from opioid availability and home care support to everyday community health promotion services, which can be client-driven thus demonstrating that such complex problems require a multipronged approach.
- The scope of the studies also varied from providing palliative and EoL care to those in need, to ‘preventive’ broader universal social change campaigns (e.g., death education)

**Box 3. Key points from gambling studies**

- Both universal and targeted approaches are employed involving various collaborations.
- Evaluations were limited. Casinos and the gambling industry were portrayed as being biased if involved in any regulation and evaluation of gambling effects and harms due to their financial dependency.

**Box 4. Key points from violence prevention studies**

- Most violence prevention approaches combined both universal and targeted strategies.
- The same named PH approach might apply different types of interventions or the aspects of the approach might be modified in some way depending on the local context.
- Collaboration and cross-sector working played important roles in nearly all PH approach to prevent/ address violence.
- Only a quarter of implementation studies provided any details about the effectiveness of the intervention/approach with less providing details on how their approach could be scaled up to other contexts.
- Detailing an intervention as ‘the most promising programme’ or an ‘adapted promising practices’ seems to bypass the need for further evaluation.
- Potential barriers to implementing public health violence prevention approaches include incompatibility of surveillance systems between regions.

### PH approaches to prevent and address ACEs

What were the PH approaches targeting/addressing? Of the eight studies which describe the application of a PH approach, seven were aimed at preventing child maltreatment, with six studies reporting both targeted and universal approaches [13–18] and one study reported on the implementation of a universal education programme in a health clinic [19]. One was aimed at preventing child sexual abuse taking a secondary and tertiary prevention approach [20]. See S1 Table for more details.

#### What were the rationales for taking a PH approach?

Six studies which reported the application of a PH approach to prevent child maltreatment cited the scale and systemic nature of the problem, the need for universal and targeted approaches, multisector collaborations and population-based approach [13–17]. The other application involving an education programme in paediatric clinics cited the need to address the drivers of maltreatment and to prevent deterioration of the child-parent relationship [19]. The Stop It Now! application aimed at preventing and stopping child abuse referred to addressing sexual abuse as every adults’ responsibility [20].

#### What were their common features?

Five studies involved using evidence-based programmes (EBPs) such as the Triple P Positive Parenting Programmes [13–16,19] with three of these PH approaches implementing more than one type of programme as part of a regional strategy [14,16,19]. Two of the studies (the Essentials for Childhood approach and the Preventing Adverse Childhood Experience: Data to Action (PACE:D2A)) reported a broad range of approaches with economic support and strategies to address social norms environments and behaviours to prevent ACEs from happening as well as reduce the impact of ACEs [17,18]. Families New South Wales (NSW) had a very similar approach but suggested further attention needed to be given to the wider determinants of family stressors [16]. Six of the studies involved multi-sector stakeholder engagement at a regional level to increase uptake from participants and endorsement across agencies [13–18]. The other two studies were more focused on addressing individual behaviours through individual counselling [19] and support by a specific service provider [20] and did not address the wider contexts in which people live and work.

#### Were there any approaches that stood out as being different from the rest?

Different approaches to ACEs prevention stood out for various reasons. One study applied their approach on a relatively small scale: 4 paediatric clinics for evidence-based programme (EBP) Period of Purple CRYING intervention [19] compared to statewide [14–18], countrywide [20] or citywide [13].

The Stop It Now! intervention used a two-stage staggered, almost triage approach, initially allowing universal coverage with a free, anonymous helpline with operators offering information, advice, support and guidance to anyone concerned about child sexual abuse which may then lead to counselling people identified as being at risk of sexually abusing children [20].

The high level of flexibility of the Triple P programme is apparent with the addition of media-based strategies for Triple P delivery to account for all preferences in Glasgow [13]. Furthermore the Triple P programme appears to have the most adaptability with 17 variants allowing to use graded reach and intensity of parenting support services [15] and could be group-based or home-based [16].

#### Did each approach provide any type of evaluation?

For clarity we have distinguished between interventions, such as the Nurse Family Partnership, the Incredible Years (IY), Strengthening Families Programme, Triple P and PURPLE which have an evidence-base, and the implementation of novel programmes. Two studies examined an evidence-based intervention as part of the PH approach [13,15] and three studies use multiple EBPs [14,16,19].

The Essentials for Childhood initiative, which is linked to the PACE:D2A cooperative agreement, [18] specifies the outcome variables and indicators to track progress over time, however no formal evaluation has been conducted as in preliminary stages of the five-year initiative [17].

The Families NSW approach reported on effectiveness evaluations of aspects of the study, including a Randomised Controlled Trial (RCT) of an intensive health home visiting service, where better maternal, child and family outcomes were reported after 30 months and greater reduction in child welfare ‘risk of harm’ reported from intervention families after 12 months [16]. Some limited qualitative data has been collected in the Stop It Now! pilot study about the helpline’s potential to influence sex abuse behaviour [20].

#### Did the study give details of how such approach could be extended?

The Stop It Now! pilot study developed online supporting documents and a toolkit, including details of the training programme, interview topic guide and call logs, for implementing a similar prevention helpline in other countries [20]. Similarly for the ‘Essentials for Childhood’ programme, the Center for Disease Control and Prevention (CDC) have published a series of technical packages, alongside a resource document which are designed to inform and guide states and communities about the best available evidence to prevent childhood abuse and neglect [17].

Those studies that implemented the evidence-based Triple P intervention reported that this could be easily scaled up, including capitalising on existing collaborations with other cities and regions that have already implemented Triple P, using a wide range of media-based population-level strategies to increase uptake, training the entire regional childcare workforce [13], and developing clear implementation guidelines [15]. Other recommendations included active involvement of targeted parenting groups, addressing cultural diversity, novel engagement strategies, and effective partnership and support by training organisation [15].

The Families NSW study highlighted the complexities of taking a regional coordinated approach to addressing ACEs and reported that extending the approach required to address existing systemic problems such as over-exclusion or under-inclusion of the population, and service capacity, delivery and orientation. The authors suggested that possible extension of the NSW reforms should include engagement and services for fathers and young people experiencing ACEs, although no specific details were given on how this could be achieved [16].

The North Caroline Institute of Medicine Task Force recommended coordinating leadership efforts, and sufficient resources and expertise to overcome barriers and maximise fidelity of implementation [14]. Furthermore, the PH approach in paediatric clinics settings highlighted the need to achieve strong buy-in from leadership and staff members at every level to support organizational readiness to successfully implement such programme [19].

### PH approaches to EoL care

#### What were the PH approaches targeting/addressing?

For the 4 studies we classified as applying a PH approach to improving EoL care, three were aimed at whole communities [21–23]. The World Health Organisation (WHO) approach reported by Leon and Colleagues aimed to improve opioid availability, increase palliative care (PC) education for healthcare workers, and include PC as a component of care in legislation within developing countries. The PH approach sought to develop cross-sector collaborations between government entities, non-government organisations (NGOs), stakeholders and health professionals. It used workshops to identify barriers to adequate opioid availability, made recommendations and influenced policy [21].

Taub and colleagues examined two community service programmes. The one in India, known as the College of Nursing Community Health (CONCH) programme, involved initialising a partnership between community health workers and volunteer village health workers nominated by community members, and supported by the Christian Medical College. CONCH is a three-tiered nurse-managed programme that promotes community health by providing direct and indirect services for all ages, including home care, clinics, health-promotion activities, school health programmes, Geriatric Club, and Counselling. By contrast, the Phinney Neighbourhood Association (PNA) Village approach in the USA features the creation of volunteer-led social support systems which are client-driven and self-governing with the aim of allowing clients at the EoL to continue living and thriving in their community [22].

The Compassionate Community Model was implemented in Canada and aims to empower primary care and non-specialist providers in the delivery of PC using educational programmes, tools and resources to enable community members to support patients and families dealing with terminal illnesses [23].

The fourth study, Improve End-of Life Care in First Nations (EOLFN), was targeted towards First Nations Indigenous communities across four specific geographical locations within Canada. Community assessments and the involvement of community elders and leaders as well as health care providers during the actual creation of the programme permitted the unique social, spiritual and cultural practices of each community to be embraced and integrated into pre-existing local health services. Importantly community members engaged with external health care organisation to address gaps in health services to better support people at the EoL and their carers [24]. See S2 Table for more details.

#### What were the rationales for taking a PH approach?

Three studies which reported the application of a PH approach to improve EoL care cited the need to develop and integrate PC into society and culture, including health care workers, the public and organisations [21,22,24]. The CONCH programme also cited the need to address the medical, psychological and social needs of older adults living with advanced life-limiting illnesses [22]. The Compassionate Communities focus was aimed at addressing the needs of patients, families (caregivers) regardless of age or disease trajectory, taking a multilevel life course approach [23].

#### What were their common features?

All of these PH approaches sought to create significant collaborations and partnerships between various organisations governmental and non-governmental entities [21], community volunteers and health workers [22,23] and community leaders and health care providers [24]. Their common aim was to bridge the gap between medicine/ health care and communities, including in contexts with limited or absent governmental funding [22].

#### Were there any approaches that stood out as being different from the rest?

One approach that stood out was the EOLFN study that developed four unique PC programmes as it recognised the significance of local conditions, local culture and local knowledge during the actual development of each programme. This community involvement right from the start of the 6 year project was reported as allowing the ‘right’ people involved on the community advisory commmitee who were heralded as influential and able to champion change [24].

#### Did each approach provide any type of evaluation?

Only one study, the PNA village approach included an evaluation of the five areas it was implemented in. The evaluation looked at whether the approach had reduced people’s social isolation, with 79% of clients stating they knew more people and 59% reporting that they felt more socially connected [22].

The WHO PH approach was deemed to be ‘successful’ as several policy changes were implemented including mandatory undergraduate course in PC at Universidad de la Sabana, Columbia and policy recommendations for the Ministry of Health regarding modifying a national policy to guarantee availability of opioid analgesics in at least one place for all 32 states; however, the impact on people at the EoL or those caring for people at the EoL was not evaluated [21].

#### Did the study give details of how such approach could be extended?

All the implementation studies reflected on whether and how the approach could be applied to other contexts. Some of the approaches provided resources and strategy documents to support wider uptake. For example, toolkits to cover core aspects of successfully initiating a Compassionate Community [23], policy documents incorporating recommendations and framework to guide policy and programme development, as well as guidelines to replicate the journey mapping process [24,25].

Details of collaborations, including national and regional competent authorities, implemented during opioid availability workshops were offered as ways of overcoming barriers which might be encountered in other countries [21]. Recommendations of how to build partnerships between volunteer-based community organisations and healthcare organisations were described in PNA Villages in USA whereby community volunteers helped connect clients, those adults living with chronic and/or life-limiting illnesses, with appropriate healthcare services and community network [22]

### PH approaches to address gambling harms

#### What were the PH approaches targeting/addressing?

Two studies described the implementation of a PH approach to reduce gambling harm. One study in New Zealand had a universal approach and described the implementation of policies, legislation and marketing campaigns to focus on community ownership of the problem [26]. The other described the implementation of recommendations by the Welsh government of both universal and targeted interventions including promoting learning module about gambling harms for General Practitioners (GPs) and providing free counselling and helpline support to people with gambling problems [27]. See S3 Table for more details.

#### What were the rationales for taking a PH approach?

The Gambling Act in New Zealand provided the rationale behind the PH approach which was in response to a rapid increase in the scale and nature of gambling in the Country [26]. The legislation and approach aimed to tackle the culture of gambling and the gambling environment. Similarly, the Welsh Government were responding to the increase in gambling-related harms, particularly for those who live in more deprived areas, with the focus on health equity for all [27].

#### What were their common features?

Both of these PH approaches focused on aspects of primary, secondary and tertiary prevention and collaborated with various third sector organisations.

#### Were there any approaches that stood out as being different from the rest?

For the Gambling Act in New Zealand to influence the *whole* gambling environment, it used the minimization framework and aimed to influence top-down from policy to intervention, health promotion initiatives and bottom-up from empowered communities to managed environments and accountability initiatives [26]. The Welsh government focused on GP eLearning modules, legislation and targeted free helpline support for people with gambling problems, without community involvement or engagement [27].

#### Did each approach provide any type of evaluation?

No specific evaluations were reported. However, for the PH approach in New Zealand, Adams and Rossen considered it not to have been successful due to a financial deal between the government and part of the gambling industry which, during these policy implementations, actually increased the industry’s profits [26]. Specifically, the Prime Minister announced a deal with the Auckland SkyCity Casino in which the Casino agreed to invest NZ$350 million in a convention centre in return for a relaxation of gambling and the doubling of profits from EGMs distributed in bars and clubs over 5 years [26].

#### Did the study give details of how such approach could be extended?

The New Zealand study highlighted the recommendation that strong, independent (not related to gambling industry) accountability was essential [26] whereas the study in Wales recommended all evidence-based player protection options. These include formal restrictions on in-play betting promotions and VIP schemes offering repeat-play incentives to large losers, along with the introduction of a mandatory levy on industry to support harm minimisation, prevention, evidence-based treatment options and research into gambling-related harm [27].

### PH Approaches to address violent behaviour

#### What were the PH approaches targeting/addressing?

Of the 19 studies identified as applying a PH approach to reducing violent behaviour, four were aimed at preventing sexual and Intimate Partner Violence (IPV) [28–31], seven focused on preventing gun [32–36] or knife violence [37,38], and three were aimed at preventing or addressing the causes of youth gang violence, including carrying weapons [39–41]. Other studies were aimed at preventing homicides, by focusing on maximising data sharing and examining patterns and factors associated with the circumstances and occurrences of violent deaths [42], and violent crimes, by developing partnerships between police, health and local government to tackle the underlying factors behind the increase in all types of serious crimes [43]. Two studies sought to prevent violence in community and public space settings [44,45], whilst a further study focused on identifying pathologically-fixated individuals [46]. See S4 Table for more details.

#### What were the rationales for taking a PH approach?

The majority of studies which reported the application of a PH approach to prevent violence cited the scale of the problem including the consequences for victims, others and family members as their main justifications for taking a PH approach. Wagman and colleagues also highlighted, that attitudes condoning Intimate Partner Violence (IPV) are widespread [30,31]. Of the nine interventions that focused on primary prevention [28–32,39,42,43,45], 6 were aimed at improving some aspects of the social determinants of health such as education and the school curriculum [28], supporting the community by establishing local community action groups [30] or promoting positive social norms [31], changing the physical environment [45] or tacking wider societal problems such as employment, personal development and housing [39] or mental ill health, education, addiction and lack of employment opportunity [43]. The latter two studies, Community Initiative to Reduce Violence (CIRV), as part of the Scottish VRU [39], and Violence Reduction Partnership (VRP) in Merseyside [43] outline a systems approach that recognises the need to impact across multiple of pathways.

#### What were their common features?

Most of the PH approaches to violence reduction used both universal and targeted strategies. Across the UK the Violence Reduction Units use similar approaches. For example the London Violence Reduction Unit (VRU) places specialist youth workers, from the Redthread charity, in Accident & Emergency departments to respond to ‘moments of vulnerability’ and intervene with young people aged 11–24 years following violent incidents, as well as promoting youth advocates programmes and major media campaigns, such as #knifefree, that are more broadly applied to the population [38]. Similarly the Scottish VRU applies a hospital-based intervention, Navigator, in emergency departments across Scotland whereby healthcare professionals focus on young people (aged 16+) to help them see the implications of knife carrying [37]. The Scottish VRU also collaborates with the charity Medics Against Violence whereby healthcare professionals lead school-based programmes to show the consequences of knife violence to pupils from different perspectives, including perpetrators, victims and families [37]. The Welsh VRU applies an Early Action Together approach, involving Public Health Wales, the country’s four police forces, police and crime commissioners, Barnardo’s and His Majesty’s Prison and Probation services, and uses early interventions and precautionary measures to support vulnerable people and examine the root causes of criminal behaviour [38]. The Merseyside VRP (previously a VRU) has focused on the partnership between clinicians, police and local government lead by an independent chairperson, with the need to benchmark data for the 10-year strategy [43]. Their strategies include Navigator, multi-media campaigns and community engagement; the approach will be subjected to ongoing evaluation against a range of indicators, such as school attendance, appearance in criminal justice system, mental health outcomes [38]. It seems that VRUs commonly apply not only media campaigns aimed at the whole population and programmes identifying populations or opportunities to intervene to prevent violence, and work with the perpetrators of violence to prevent re-offending.

Three studies reported approaches which focused solely on those at risk of perpetrating violence [32,39,46]; One USA study considered the high risk group to be African American men aged 18–35 years and sought to intervene when they attended routine medical appointments [32], whereas another identified high-risk offenders of physical violence and weapon carriage from police intelligence systems [39] and in another study member of staff from a Fixated Threat Assessment Center identified people having a profile of concerning behaviour [46]. Once the target population was established, each of these approaches applied different interventions such as GP counselling [32], diversionary activities including personal development and job-readiness [39], or evidence-based interventions to deactivate some of the drivers of violence [46].

Five other studies focused on environments at high-risk of violence, either neighbourhoods with the highest level of homicides and non-fatal shootings [33,36,40,41] or large public institutions where family disputes and violence are more likely to occur. [45]

#### Were there any approaches that stood out as being different from the rest?

Some of the PH approaches to violence prevention focused on individuals deemed to be at risk, for example, the preventive counselling sessions for gun violence delivered by physicians during routine clinical encounters with high-risk males [32] and a collaboration, using the case management system by a Fixated Threat Assessment Center to identify subgroups of individuals who maybe at high-risk of carrying out grievance-fuelled violence [46].

Other PH approaches aimed to affect the wider conditions that were considered important for the intervention to work and be successful. For example, the Community Violence Prevention Plan (CVPP) focused on cross-sector data sharing to engage stakeholders to develop solutions to commonly reported barriers [44]. Similarly, the development of partnerships and coalition building and the co-creation of programmes was the main focus of the violence prevention alliance of the WHO [36]. Other approaches included the development of the National violent death reporting tools [42] and surveillance systems compatible between schools [35].

The various scale of the approaches was noticeable. For example, the DELTA FOCUS included 14 USA states in the coalition [31], compared with a single city focus (Wilmington in the USA state of Delaware) involved in Violence Prevention Alliance [36]; similarly, the creation of a Violence Reduction Unit at regional levels in England compared to a national approach in Scotland. The long timespan for certain PH approaches was also evident, with a 10-year strategy by Merseyside VRP [43] and a 20-year span for the CV and hotspot policing simulation modelling techniques [40].

Several of the approaches used the CV intervention, however there was variation in its application. The Safe Streets programme, based on CV, in Baltimore did not include the usual specialised ‘violence interrupters’ to mediate conflict, but assigned this task to street workers [33]. The Chicago’s Ceasefire model in Crown Heights included the additional community mobilization campaign [34] and the CVPP used CV with violence interrupters as well as a hospital-based screening and case management intervention aimed at identifying individuals at risk of violent behaviour [44].

Some adaptability was apparent in the Rape Prevention and Education (RPE) programme in North Carolina in which 3 regions of the state received differing strategies to change attitudes and behaviours in relation to sexual violence prevention according to their community needs [28].

There was also a difference in focus for the two Domestic violence prevention Enhancement and Leadership through Alliances (DELTA) implementation studies. One, DELTA programme, Preparing and Raising Expectations for Prevention (PREP) involved an online document support system for project staff to record inventories of organisational changes and prevention activities actioned within each coalition which had 5 regional coaching hubs to provide training and support [29]. Whilst the later study DELTA Focusing on Outcomes for Communities United with States (FOCUS), used a chronic disease management system, CD-MIS, to track and facilitate programme evaluations and implemented ‘Project PIN (Performing, Informing Norming)’, which was aimed at creating community-relevant messaging to promote social norms, alongside other prevention strategies such as the Bystander approach [31].

The Wakanheza Projects stood out as using the most culturally sensitive approach; the approach has been implemented in several large public places, aiming to promote healthier communities by altering the physical environment [45]. The project was cross-sector and aimed to identify implementable strategies to create more welcoming environments in public spaces, intending to reduce stress for parents, children and teenagers and hence violent behaviour.

#### Did each approach provide any type of evaluation?

Of the 19 studies, only five reported outcomes linked to a reduction in violent crime and effectiveness of their approach [33,34,37,39,41], with Baltimore Safe Streets, the Scottish VRU and Save Our Streets in Crown Heights reporting a reduction in the numbers of homicides and shootings [33,34,37]. The CIRV study reported a reduction in the carrying of weapons, principally knives [39] and the Safe Street Baltimore study by Tibbs and colleagues reported that 55% of mediations occurred before conflict escalated [41]. Other evaluations captured system indicators of change.

The DELTA PREP study reported an increase in the 10-item index of the Prevention Capacity of the organization [29]. Cerda and colleagues provided results about CV plus ‘hot-spot’ policing using simulation modelling techniques to predict the potential increase or decrease in homicides over 20 years [40].

Two other studies provided very limited results: the preventive counselling by GP reported results from post encounter interviews [32] and The Wakanheza Project provided some findings of immediate impact on physical environment, organisational and staff culture [45].

The VRUs in London and Wales referred to the pioneering VRU in Scotland as an evaluation [38] and two other studies indicated that they applied the ‘most promising programmes’ but did not include an effectiveness evaluation [28,30].

Several studies highlighted the difficulties of evaluating violence reduction interventions; for example, the K-12 Schools Security study highlighted the challenges in assessing the effectiveness of school gun violence interventions citing the differences in comparable data captured by difference surveillance systems [35]. Other studies also highlighted the evaluation challenges of sharing data across organisations, proposing that engaging all stakeholders in the approach from the onset [44] as well as the development of a national violent death reporting tool which links related data from various sources and agencies’ [42] could generate solutions.

#### Did the study give details of how such approach could be extended?

Three studies did not reflect on whether or how the approach could be applied to other contexts [28,32,39]. Some of the other approaches provided resources and strategy documents to support wider uptake. For example, the VRU in Scotland has a collection of easily accessible online resources and tools giving practical advice for implementing a PH approach to violence and other projects that they are currently implementing as PH approaches [37].

Other studies suggested to be mindful of when developing a PH approach, with the CDC Youth Violence Prevention Centres (YVPC) proposing that researchers work to construct “packages” of effective interventions, help communities to understand the role and requirements of evidence-based practice, carefully select programmes and cultivate capacity (both innovation-specific and general organizational), and coordinate and align efforts within the community [36]. The DELTA PREP programme highlighted that a PH approach needs to be flexible and adaptive and included eight support areas to promote organisational change and develop action plans and prevention activities for the domestic abuse coalitions (each one varying in size, budget and context) [29]. The rapid feedback programme improvement process used within the DELTA PREP identified how coalitions perceived the usefulness of project support and allowed adjustments based on participant feedback and needs [29].

Other recommendations for upscaling and roll out of approaches include the Safe Homes And Respect for Everyone (SHARE) Project by Wagman and colleagues which reported seven recommendations for wider roll out of their IPV prevention approach, including the careful selection and training of project staff, the promotion of community ownership, and the need to establish partnerships with key people and groups in intervention regions [30]. The Wakanheza Project was implemented in multiple settings, took into account the individual organisational cultures and acted on building collective efficacy across the settings. The three large urban community organisations, provide some evidence-based requirements (initiating events, initial implementation, changes in physical environment and organisational culture) for starting up a Wakenheza Projects in other organisations [45]. Other studies reported the need for the underlying evidence-base of interventions to be the most relevant, complete and easily accessible for others to use [42,44].

### Synthesis of implemented PH approaches

Across the four categories several aspects of a PH approach are consistently operationalised. For all the categories the complex nature of the issue necessitated a PH approach with violence prevention, preventing and addressing ACEs and preventing and addressing gambling related harms also citing the scale of the issue as a further underpinning rationale. Most had aspects of primary, secondary and tertiary prevention, using universal and targeted approaches. Most involved multisector collaborations, although a few focused on healthcare professionals (e.g., for ACEs: clinic staff members were trained to deliver PAP interview techniques to parents [19]; e.g., for violence: GPs to counsel patients about gun violence [32]) as deliverers of the PH approach.

We looked to see whether the approaches had evolved over time. Whilst there was some evidence for some of the well-known programmes such as CV and Essentials for Childhood having additional aspects of the approach, there was no overall pattern of the approach per se evolving from an individual to a system focus.

Across the four areas few approaches focused on the wider determinants of health as a means of creating the conditions for health and addressing the drivers of behaviours associated with the problem; PH approaches to support EoL care differed from the other categories as the implemented approaches were aimed across the life course, creating the conditions for living and dying well. Most of the approaches were responsive and adaptive to the local context, having an awareness of socio-cultural norms which influenced who and how the programme was delivered.

The evaluations of PH approaches highlight the issues of trying to capture system level changes which impacts on individual behaviours. None of the evaluations, across the four areas, captured the impact on individual behaviour, presumably reflecting the time period between creating system level change and individual level outcomes. Some of the evaluations sought to assess proxy outcome measures, theorising that these were important determinants of addressing ‘risky’ behaviours.

## Discussion

This scoping review sought to chart the diversity and nature of the issues for which a PH approach is advocated over the last decade; to undertake a deep dive into four societal issues where a PH approach is being used to understand whether and how such approach has been operationalised and evaluated including any proposals for widespread adoption and scale up.

### Categorization of issues where a public health approach is being described

The results from this scoping review show the extent to which health and societal issues have been linked to a PH approach identifying over 750 different issues which called for a PH approach which we grouped into 28 overarching categories across a wide geographical spread. Further exploration to understand the rationale behind the need for a PH approach for these issues is merited.

### Chart the nature of the descriptions and applications of PH approaches across and within four purposively sampled categories of issues over the last decade

The focus on four purposefully sampled subject categories allowed us to explore whether the nature of studies proposing or describing a PH approach had changed over time, in particular whether there was any trend in publications calling for a PH approach to address an issue to more descriptions and applications of the approach in subsequent years. Over the last decade there has been a gradual increase in the number of applications of a PH approach, although these publications were only 13% of the studies.

Although we expected to see an increase in the number of studies describing and applying a PH approach across our four categories over time, there was no consistent pattern.

### Capture any evaluations and descriptions as to how it can be scaled up

Another noticeable feature is the lack of follow-up and evaluations of the effectiveness of the PH approaches being implemented, although several studies reported the use of an ‘evidence-based’ approach, which perhaps reduced the need for an evaluation. The exception to this were the studies addressing and reducing violent behaviour. A possible reason for this, is that criminal and violent assaults are, and have been, recorded within the police force and criminal system; thus, some baseline data is readily available which can be tracked over time to monitor any differences in trends where the approach is being implemented.

For some recent ACE’s studies that are in the preliminary stages, e.g., Essential for Childhood programme and Preventing Adverse Childhood Experiences: Data to Action, the outcomes measures have been factored in, according to the time span of each study and in relation to how long any effect of the approach will take before it is possibly affecting any primary outcome variables.

When PH approaches involve so-called evidence-based interventions, or the ‘most promising programmes’, there is a need to consider the culture, the local environment and context that it is being applied to, as well as maximising the readiness and capacity of all the organisations involved.

The potential for scaling up and across of PH approaches was considered in some studies with the handbooks, resources and toolkits used during implementation being made available (VRUs [37]; Compassionate Community [23]; Triple P [15]; Stop It Now! [20]; Essentials for Childhood [17]; PACE:D2A [18]; EoL care [24]). These frameworks outline the principles and ways of working and have been created to allow for adaptation to the local context.

Noticeably one study included strategies to maximise the adaptability of the approach in its implementation rather than for future scale up. The EoL care in First Nations used a participatory action research approach which allowed for the unique socio-cultural aspects of each individual community to be incorporated in the development of the programme. This was followed through in the amount of available recommendations and documentations for future adaptations to other First Nations communities although limited evaluation was reported [24].

### Are PH approaches responding to calls for a fifth wave in public health?

From our discussion we have highlighted some of the main similarities and differences between how PH approaches have been applied across and within four societal issues. Hanlon and colleagues describe a challenge to PH as to how it responds to the nature of the issues affecting health and wellbeing, proposing the need for a new PH approach and suggest some of the characteristics and qualities such an approach might entail [2]. This complements and extends other approaches such as ‘One Health’ [47], Planetary Health [48], ecological approaches [49]. It recognises the systems which generate ill health as complex and adaptive, with interdependencies and positive and negative feedback loops [50] and thus attempts to change the systems behaviour needed to involve a shared understanding of the nature of the issue and cooperative ways of working to address it. There were aspects of the qualities in some of the approaches, particularly in EoL care PH approaches which emphasise cross-sector collaborative working and adaptive, culturally responsive frameworks. As Hanlon and others [51] suggest, how we understand the nature of the problem determines how we respond, and further exploration of the other categories to determine the rationale for a PH approach is merited.

### Limitations

We acknowledge several limitations with this scoping review. Firstly, although very wide, our inclusion criteria did not include public health “frameworks” or other descriptors such as “population health” approaches. This was because we were interested in the description and application of a “public health approach” and so only searched for studies with these terms. Secondly, we acknowledge the overlap of some of the issues which could have been categorised in more than one category. In determining which category we identified the primary focus of the issue for each included study by using two reviewers when necessary. Thirdly, for the deep dive and narrative synthesis we have only focused on 4 specific subjects’ areas due to the large volume of studies and time constraints, and we acknowledge that the other subject areas may define and operationalise significantly different PH approaches to the ones described here.

Finally, as this is a scoping review, we have not evaluated of the quality of included studies. We have provided a comprehensive PRISMA checklist of our scoping review process and considerations as S1 Checklist.

## Conclusion

In conclusion we have described the range and extent of health and societal issues where a PH approach has been implicated within studies spanning over a decade. We have added to the knowledge regarding the development of PH approaches which we anticipate will continue to grow. Our review has examined four issues in detail and found that only a small percentage of publications reported on how the approach had been operationalised. We described these PH approaches, examining common features and differences, and highlighted some adaptability and scale-up strategies. However, despite the rise in calls for PH approaches few of the studies gave details of evaluation results.

Further work involves examining any evaluation results published from included studies that were still in early stages of implementation and examining the other societal issues identified in this review to understand the rationale for a PH approach, the characteristics of the approach and any outcomes reported from its implementation.

## Supporting information

S1 Checklist PRISMA ScR checklist. (DOCX)

S1 Table Adverse childhood experiences studies. (DOCX)

S2 Table End of life care studies. (DOCX)

S3 Table Gambling studies. (DOCX)

S4 Table Violence studies. (DOCX)
