# Supplementary material for: Exploring the rise and diversity of health and societal issues that use a public health approach: A scoping review and narrative synthesis
Source: PLOS Glob Public Health. 2024 Jan 10;4(1):e0002790. doi: 10.1371/journal.pgph.0002790 (PMC10781110; doi:10.1371/journal.pgph.0002790)
Supplement: S1 Table — (DOCX) [file pgph.0002790.s002.docx]

S1 Table: Publication details of those included studies that reported applying a public health approach to Adverse Childhood Experiences, with details of how each has been operationalised (n=8)

| **Study** | **Application or intervention name(s) with location** | **Aim of the intervention** | **Rationale for PH approach** | **Nature of the abuse/ neglect** | **Focus on Primary/secondary/tertiary prevention?** | **Anticipated impact (individual, family, neighbourhood)?** | **Population targeted or universal level?** | **Cross-sector working/ collaboration** | **Evaluation done? If so, what results** | **How they have attempted to deliver at scale** |
| --- | --- | --- | --- | --- | --- | --- | --- | --- | --- | --- |
| Barlow & Calam (2011) [1] | Triple P (Positive Parenting Program) part of integrated workforce approach Glasgow, UK | ensure all parents are able to develop the skills they need to parent effectively and use existing policy frameworks to move children on to higher levels of service provision where necessary. | Scale and systemic nature of the problem,  Prevent future ill health,  Economic case for early intervention | Child protection | Primary, secondary and tertiary | Individual, family level (no mention of SDH) | Tiered approaches with universal parenting advice (level 1) and targeted interventions (2-5 levels) | collaboration with Amsterdam, related media technology and businesses;  with families | evidence-based intervention and refers to RCT results reducing population-level indicators of maltreatment* | Collaboration with other regions that implemented Triple P;  Use media-based strategies for program delivery to account for all preferences; |
| Richmond- Crum et al. (2013)[2] | North Carolina Institute of Medicine (NCIOM) Task Force recommendations, USA including Triple P, Period of PURPLE crying®: Keeping Babies Safe in North Carolina, Nurse Family Partnership, The Incredible Years parenting program, and Strengthening Families Program 6-11 | Promote better coordination between partners health, family development and community support of families and move from a 'child welfare' frame of child maltreatment to a PH upstream investments frame | Protecting and improving health of individuals and communities | Child maltreatment protection | Primary (using the socio-ecological framework) | Creating healthy environments, by federal, state, and local PH  agencies work together to prevent harm to children before it can occur through programs and prevention strategies directed at children, families, and the environment in which they interact. | Universal and targeted interventions | State-level leadership coordinated by NC Division of DPH and Prevent Child Abuse NC (PCANC) to develop and oversee prevention efforts across public and private agencies; | Not undertaken; reported ‘increasing use of EBPs’ | Recommends ensuring coordinating leadership, sufficient resources and expertise to overcome barriers and maximise fidelity of implementation.  Developed shared indicators, common grant requirements and shared evaluations, reducing duplication of effort and gaps in services; maximise fidelity of the EBPs. |
| Sanders and Kirby (2014) [3] | Triple P system with 5 level/ intensities with 17 program variants in USA | Improve social, emotional and behavioural outcomes for children; in the most cost-effective and time-efficient manner; | Scale of the problem, need for a wider reach | Parenting support for children’s wellbeing | Primary, secondary and tertiary | ‘an approach that emphasizes the targeting of parents at a  whole-of-population level, utilizing a blend of universal and targeted interventions, to achieve meaningful change in population-  level indices of child and parent outcomes’ | Universal and targeted approach using graded reach and intensity of parenting and family support services | Partnerships between partner organisations that support the implementation of Triple P program variants; | Given as EBP and reported results of meta-analysis that includes 101 studies including 62 RCTs [4];  after 2.5 years counties randomly assigned to Triple P had lower rates of child maltreatment on many indices* | Development of implementation guidelines aimed at organisations;  involvement of target parenting groups; addressing cultural diversity and novel engagement strategies; effective partnership and support by training organisation. |
| Horn et al. (2015) [5] | Stop It Now! (pilot study) in UK and Netherlands | mobilise adult families and communities to take actions that protect children before they are harmed | ‘All adults are responsible for preventing child sexual abuse (CSA)’. | CSA prevention | Secondary and tertiary | Affecting behaviour of perpetrators with  Individual therapeutic sessions by an outpatient centre | Universal and targeted helpline for *anyone* concerned about CSA; | Collaboration between senior practitioners, from Stop It Now! charity, and helpline operators including academic graduates or other professional backgrounds from the police, probation, social work and psychology. | some limited qualitative data about helpline’s potential to influence users’ behaviour | Provision of a toolkit, including online documents for each component, for developing prevention helplines in other European countries  Draws on experience of other similar hotlines |
| Churchill and Fawcett (2016) [6] | Families NSW (mixture of EBP including Triple P and Brighter Futures) and universal home visiting support;  Keep Them Safe Strategy NSW, Australia | prevent child maltreatment and promote child welfare by improving the coordination and integration of services, and strengthen community support for families and re-orientate frontline practice towards holistic, preventative, child centred and family-focused approaches | Scale of the problem (increasing child protection cases), recognising the need for service and policy reform | Child maltreatment | Primary, secondary and tertiary | Impact on families – recognised that a limitation was not affecting the complexity of people’s lives | Universal (health home visiting services to all new mothers within two weeks of giving birth; maternal mental health screening; Triple P as group-based, home-based or multi-component basis) and targeted interventions | cross-departmental initiatives;  Programs outsourced and delivered by state child welfare and third sector services;  inter-agency working and multi-agency relationships to promote new referral pathways and service provision | some effectiveness evaluations, Brighter Futures study RCT study of intensive health home visiting service with maternal, child and family outcomes: compared to controls after 30 months: Mothers had better mental health scores, children better cognitive development and mental health at 18 months;  In comparison group there were greater reduction in child welfare ‘risk of harm’ reports among families engaged with the programme for 12 months or more, and fewer placements of children in state care. | Incorporating a broad and varied range of interventions targeting universal and specific age groups; Combining such a variety of interventions allows delivery to be responsive and relevant to the needs of the population. |
| Nong et al. (2020) [7] | Period of PURPLE Crying, social and economic stressor questionnaire and Parenting Action Plan (PAP) Houston Texas, USA | help parents, increase knowledge of common infant issues, set realistic expectations and address family stressors | impact the underlying drivers of maltreatment and reduces the deterioration of the parent-child relationship | Child Maltreatment | Primary and secondary | Impact was on implementation of the programme and acceptability to mothers (individuals) | Universal for all maternal caregivers at newborn well-infant visits | Collaboration between clinic staff members and the intervention trainers to deliver PAP | Implementation evaluation with no child outcomes;  PURPLE is EBP;  PAP is based on Triple P and SafeCare, which is given as a child maltreatment prevention program; | Organisational readiness - needs strong buy-in from clinic leadership and staff members;  Several modalities in training motivational interviewing to cater varying experience levels; understand organisational and structural strengths of each clinic. |
| Ottley et al. (2022) [8] | Essentials for Childhood Initiatives in 7 states in USA | Strengthening financial supports and changing social norms | Scale of the problem and focus on comprehensive and multisector efforts that support environments and provide access to social services, health care, and employment | Child abuse and neglect (CAN) | Primary (focusing on the social and economic conditions ie wider SDH) | Essentials focus on 2 strategies from the CAN technical package: strengthening economic supports and changing social norms.  These strategies are intended to impact the social conditions that put children at-risk or protect  them from CAN | Broad range of prevention strategies (Universal and targeted – Temporary Assistance for Needy Families TANF) | All states used existing multisector coalitions^Ψ^ to form public health partnerships with states & to engage in outreach activities, mobilize public support, promote the state’s activities, and unite diverse interests | Evaluations involved program-level outcomes which varied across states; list of outcomes and indicators given but no results. | Centers for Disease Control and Prevention (CDC) have published a series of technical packages, alongside a resource document to address CAN and ACEs which are designed to inform and guide states and communities about the best available evidence to prevent CAN |
| Guinn et al. 2022 [9] | Preventing Adverse Childhood Experiences: Data to Action (PACE:D2A) cooperative agreement  designed to leverage multisector partnerships and resources. Initiative by CDC’s Division of Violence Prevention, USA | create a state-wise surveillance infrastructure that ensures the capacity to collect, analyse and use ACE data to inform statewide prevention strategies and activities;  implement at least 2 prevention strategies based on the best available evidence; | PH considers the conditions necessary to  assure the health, safety, and well-being of entire populations. | CAN | Primary eg. Ottley as well as secondary through programmes | State-wide indicators and engagement in programme (family) indicators  Most outcomes are measured at the community and societal levels and focus on risk and  protective factors for ACEs. | Universal and targeted: Multigenerational strategies and approaches based on the best available evidence [10] | Main focus on leveraging multisector partnership and resources to: enhance and build infrastructures for state-level data collection, implement at least 2 ‘best-available’ prevention strategies and undertake data to action activities | Ongoing evaluation (3 years of funding from 2020) | Published suite of technical packages which outlines several strategies and approaches to prevent and mitigate the harms of ACEs;  Also CDC’s capacity assessment tool and Preventing Adverse Childhood Experiences Trainings modules and resources widely accessible online for healthcare and education professionals^$^ |

*Significant reductions in reported hospitalizations and injuries due to child maltreatment, out-of-home placements and number of founded cases of child maltreatment. [11]

Ψ Sectors from community-based organizations, businesses, health centers, academic institutions, child advocacy organizations, child welfare, education, early childhood, housing, health and human services, and other state and local agencies (S19);

$ <https://vetoviolence.cdc.gov/apps/main/aces-resources>

Abbreviations: ACEs Adverse Childhood Experiences; CAN child abuse and neglect; CDC Centers for Disease Control and Prevention; CSA child sexual abuse; EBPs evidence-based programmes; PAP Parenting Action Plan; PH Public Health; SDH social determinants of health; UK United Kingdom; USA United States of America;

References

1. Barlow J, Calam R. A Public Health Approach to Safeguarding in the 21st Century. Child Abuse Review. 2011;20(4):238-55. doi: 10.1002/car.1194

2. Richmond-Crum M, Joyner C, Fogerty S, Ellis M, Saul J. Applying a public health approach: the role of state health departments in preventing maltreatment and fatalities of children. Child welfare. 2013;92(2):99-117.

3. Sanders M, Kirby J. A public-health approach to improving parenting and promoting children's well-being. Child Development Perspectives. 2014;8(4):250-7. doi: 10.1111/cdep.12086

4. Sanders M, Kirby J, Tellegen C, Day J. The Triple P-Positive Parenting Program: a systematic review and meta-analysis of a multi-level system of parenting support. Clin Psychol Rev. 2014;34(4):337-57.

5. Horn JV, Eisenberg M, Nicholls CM, Mulder J, Webster S, Paskell C, et al. Stop It Now! A Pilot Study Into the Limits and Benefits of a Free Helpline Preventing Child Sexual Abuse. Journal of Child Sexual Abuse. 2015;24(8):853-72. doi: 10.1080/10538712.2015.1088914

6. Churchill H, Fawcett B. Refocusing on Early Intervention and Family Support: A Review of Child Welfare Reforms in New South Wales, Australia. Social Policy and Society. 2016;15(2):303-16. doi: 10.1017/S1474746416000038

7. Nong YH, Lopez KK, Mandell D. Implementation Evaluation of an Education Program in Pediatric Clinics. Journal of Applied Research on Children. 2020;11(1).

8. Ottley PG, Barranco LS, Freire K, Meehan A, Shiver A, Lumpkin C, et al. Preventing Childhood Adversity Through Economic Support and Social Norm Strategies. American Journal of Preventive Medicine. 2022;62(6):S16-S23. doi: 10.1016/j.amepre.2021.11.016

9. Guinn A, Ottley P, Anderson K, Oginga M, Gervon D, Holmes G. Leveraging Surveillance and Evidence: Preventing Adverse Childhood Experiences Through Data to Action. American Journal of Preventive Medicine. 2022;62(6):S24-S30. doi: 10.1016/j.amepre.2021.12.003

10. Centers for Disease Control and Prevention. Preventing Adverse Childhood Experiences: Leveraging the Best Available Evidence. Atlantia, GA: National Center for Injury Prevention and Control, Centers for Disease Control and Prevention; 2019.

11. Prinz R, Sanders M, Shapiro C, Whitaker D, Lutzker J. Population-based prevention of child maltreatment: the U.S. Triple p system population trial. Prev Sci. 2009;10(1):1-12. doi: 10.1007/s11121-009-0123-3
