# Supplementary material for: Exploring the rise and diversity of health and societal issues that use a public health approach: A scoping review and narrative synthesis
Source: PLOS Glob Public Health. 2024 Jan 10;4(1):e0002790. doi: 10.1371/journal.pgph.0002790 (PMC10781110; doi:10.1371/journal.pgph.0002790)
Supplement: S2 Table — (DOCX) [file pgph.0002790.s003.docx]

S2 Table: Publication details of those included studies that reported applying a public health approach to End of Life Care, with details of how each has been operationlised (n=4)

| **Study** | **Application/ intervention name(s)/ Location** | **Aim of the intervention** | **Rationale for PH approach** | **Nature of End-of-Life care** | **Focus on Primary/secondary/tertiary prevention?** | **Anticipated impact (individual, family, neighbourhood)?** | **Population targeted or universal level?** | **Cross-sector working/ collaboration** | **Evaluation done? If so, what results** | **Details of how scalable to other context/ what recommendations** |
| --- | --- | --- | --- | --- | --- | --- | --- | --- | --- | --- |
| Leon et al (2011) [1] | World Health Organisation (WHO) Public Health (PH) approach, Colombia | improve opioid availability, increase palliative care (PC) education for healthcare workers; include PC as a component of care in legislation | Integrate PC into society: availability and access to PC and to drugs; appropriate policies; education of health workers and the public; and implementation of services | Opioid availability, education of healthcare workers, inclusion in legislation and policy | Primary, secondary and tertiary; both universal and targeted | Multilevel impact (individual, healthcare service, wider public, other developing countries) | Universal (developing countries) | collaboration between government entities, non-governmental organizations (NGOs), stakeholders and health professionals to diagnose the problems and barriers in the availability and access to opioids, as well as identify solutions. | some research outcomes triggered some changes: During opioid workshop, participants identified barriers to adequate opioid availability in their regions and propose solutions to overcome those barriers leading to new regulation mandate.  Medical school survey resulted in a mandatory undergraduate course in PC & preliminary policy recommendations were drafted. | The initial collaboration workshop with pain and PC specialists and the workshop objectives identified individual barriers to adequate opioid availability in each region and propose solutions to overcome those barriers. These are given as possible suggestions for implementing similar initiatives in developing countries. |
| Tompkins (2018) [2] | Pallium Canada adopts Compassionate Community (CC) model (through education, knowledge mobilization, project facilitation and leadership), Canada | Health Promoting PC to form policy and practice coalitions of support for everyone affected by end-of-life events | Focuses on physical, cultural, psychological, social, and spiritual needs for patients and families (caregivers), regardless of age or disease trajectory | supporting communities:caregiving, dying, death, and grieving are everybody’s responsibility | Primary, secondary and tertiary; both universal and targeted | Multilevel impact (individual, healthcare service, wider public) | Universal (social change in 13 community areas; death education, inter-professional health care) | faith communities and movements to reduce stigma around ageing and dementia (Age Friendly Communities and Dementia Friendly Communities). | Not yet: Evaluating volunteer-led projects operating within the CC model has been a challenge. Because many initiatives are not-for-profit and volunteer based, funding and resources allocated to develop evaluation tools has been limited. The 13th area of focus in CCC, mandates evaluations to measure the success and usefulness of CC initiatives to justify their presence, their funding, and their support. For this reason, Pallium in partnership with CC initiatives across the country, is applying for funding to develop these tools, which will allow them to assess project performance. | Compassionate Care Charter (CCC) reported as a best practice framework as expandable and flexible for creating a Compassionate Community in any setting;  Applying the lens of caregiving, dying, death and grieving to existing initiatives- quicker spread & increases sustainability;  Mobilising CC: Toolkit to cover core aspects of successfully initiating a CC; Engagement events for community members and stakeholder allowing CC initiatives to come together; |
| Kelley et al (2018) [3] | Improve End-of-Life Care in First Nations Communities EOLFN. (developing PC programs and creating a culturally appropriate theory of change to guide program and policy development), Canada | Improve first nations communities’ formalized local PC programs, improve access to medical services, especially pain and symptom management. | Use of participatory action research PAR; community capacity development and an integrative (social, spiritual, cultural etc.) framework to (co)create PC program. | provide a Canadian example of implementing a public health approach to PC in an Indigenous context using PAR. Provide evidence of the effectiveness of a community capacity development as a strategy and illustrate how to implement it. | Primary, secondary and tertiary; targeted to Indigenous populations | Multilevel impact (individual, healthcare service, wider public) | Targeted (4 first nation populations) potential to be adopted to indigenous communities elsewhere in Canada and internationally) | Community assessment, Elders and Knowledge Carriers, community leaders and First Nations health care providers created PC programs grounded in the unique social, spiritual and cultural practices of each community, and integrated them into local health services | uses Kelley’s Developing Rural PC model as conceptual framework; and followed Prince and Kelley’s Integrative Framework for Conducting Research with First Nations communities | Two policy documents were created based on this research “Recommendations to Improve Quality and Access to Palliative Care in First Nations Communities”; “Framework to Guide Policy and Program Development for PC in First Nations Communities”  Also includes overarching keys to success/recommendations from all 4 cases |
| Taub (2019) [4] | 2 community service programmes:  College of Nursing Community Health (CONCH) programme in Vellore, India (low-resource country);    Phinney Neighbourhood Association (PNA) Village in Seattle, USA (high-resource country) | Foster partnership between healthcare organisations and community to expand community-based services for ageing populations | Lack of government funding.  The CONCH programme successfully integrates community health nurses with volunteer village health workers to address the medical, psychological, and social needs of older adults living with advanced life-limiting illness.  The PNA Village mobilises community volunteers to provide basic social support for clients with advanced illness but does not directly partner with the healthcare system. | Describes two organisations in two different countries that provide health and social services to community-dwelling adults with advanced illness. Promote increased partnerships between healthcare organisations and community volunteers to support a public health approach to community-based PC. | Mostly tertiary | Individual and familial level | Universal (community dwelling adults with advanced illness) | CONCH programme: Partnership between community health workers and volunteer village health workers (VHW), nominated by community members and supported by the Christian Medical College;  Although no such partnerships were initialised in PNA as volunteer-led social support for clients with advanced illness it was given that some 25% of PNA Villages have established partnerships with healthcare or social service agencies;  Neither program rely on government funding. | Some limited qualitative evaluation reported: for 5 PNA villages aimed at reducing client’s social isolation: 79% of clients felt they knew more people and 59% felt more socially connected. | PNA Village offers formal and informal methods of feedback for continued improvement. Since it is client driven and independently organised, it can adapt transparently and fluidly to change.  Recommends increased collaboration and partnerships between volunteer-based community organisations and healthcare organisations in the US to provide improved services to and offset costs for community-dwelling adults with advanced life-limiting illness. |

Abbreviations: CC compassionate community model; CCC Compassionate Care Charter; CDC Centers for Disease Control and Prevention; CONCH College of Nursing Community Health; EoL end of life; EOLFN Improve End-of-Life Care in First Nations Communities; NGO non-governmental organizations; PAR participatory action research; PC palliative care; PH Public Health; PNA Phinney Neighbourhood Association; SDH social determinants of health; UK United Kingdom; USA United States of America; VHW village health worker; WHO World Health Organisation;

References

1. Leon M, Florez S, De Lima L, Ryan K. Integrating palliative care in public health: the Colombian experience following an international pain policy fellowship. Palliative medicine. 2011;25(4):365-9.

2. Tompkins B. Compassionate Communities in Canada: it is everyone's responsibility. Annals of palliative medicine. 2018;7(Suppl 2):S118-S29.

3. Kelley M, Prince H, Nadin S, Brazil K, Crow M, Hanson G, et al. Developing palliative care programs in Indigenous communities using participatory action research: a Canadian application of the public health approach to palliative care. Annals of palliative medicine. 2018;7(Suppl 2):S52-S72. doi: 10.21037/apm.2018.03.06

4. Taub J, Coats H, Coats C, Doorenbos A, Siva R, Sadan V. Palliative care delivery: descriptions of community-based services in Vellore, India, and Seattle, Washington. International Journal of Palliative Nursing. 2019;25(1):39-45. doi: 10.12968/ijpn.2019.25.1.39
