# Supplementary material for: Exploring the rise and diversity of health and societal issues that use a public health approach: A scoping review and narrative synthesis
Source: PLOS Glob Public Health. 2024 Jan 10;4(1):e0002790. doi: 10.1371/journal.pgph.0002790 (PMC10781110; doi:10.1371/journal.pgph.0002790)
Supplement: S3 Table — (DOCX) [file pgph.0002790.s004.docx]

S3 Table: Publication details of those included studies that reported applying a public health approach to gambling harms, with details of how each has been operationlised (n=2)

| **Study** | **Application/ intervention name(s)** | **Location** | **Aim of intervention** | **Rationale for the PH approach** | **Focus on primary/secondary/tertiary prevention?** | **Anticipated impact (individual, family, neighbourhood)?** | **Population targeted or universal level?** | **Cross-sector working/ collaboration** | **Evaluation done? If so, what results** | **Details of how scalable to other context/ any recommendations?** |
| --- | --- | --- | --- | --- | --- | --- | --- | --- | --- | --- |
| Adams et al. (2012) [1] | Passing of the New Zealand Gambling Act in 2003 | New Zealand | To tackle the whole gambling environment ‘to control the growth of gambling’,  ‘to prevent and minimise the harm caused by gambling’ ‘to ensure that money from gambling benefits the community’ and ‘to facilitate community involvement in decisions | In response to a shift from low-potency non-continuous forms of gambling to high-potency continuous forms; rapid increase in consumption which treatment alone could not address | Primary, secondary and tertiary (using adapted harm minimising framework: top-down & bottom-up initiatives) | Individual, neighbourhood | Universal by policies and legislation, marketing campaigns focused on community ownership of problem | a number of organizations—such as Hapai Te Hauora Tapui, the Salvation Army and the Problem Gambling Foundation—undertook a range of initiatives that involved working alongside communities in raising awareness of gambling issues and in supporting ways for them to manage harm from gambling | None given;  (A network of vested interests was given as reason for lack of success) | Requires strong and independent accountability; |
| Atherton (2020) [2] | Welsh Government response to the Chief Medical Officer for Wales 2017 annual report | Wales | ‘improve quality of life for all and to achieve health equity.’ | PH action to reduce harm from gambling should not focus solely on individuals but should include a wide range of measures including advocacy, information, regulation and appropriate prohibition in a coordinated way | Primary, secondary and tertiary | Individual, neighbourhood | Universal (GP eLearning module) and targeted (Healthy settings programme - free counselling, training and helpline support to people with gambling problems) | Welsh government engaged with GambleAware & Royal Society for PH eLearning module for GPs; | None given; | Recommendations: All evidence-based player protection options should be implemented along with the introduction of a mandatory levy on industry to support harm minimisation |

Abbreviations: GP general practitioner; PH Public Health;

References

1. Adams P, Rossen F. A tale of missed opportunities: pursuit of a public health approach to gambling in New Zealand. Addiction. 2012;107(6):1051-6.

2. Atherton F. Gambling-related harm and the public health approach: addressing the challenges in Wales. Public health. 2020;184:60-2.
