# Supplementary material for: Exploring the rise and diversity of health and societal issues that use a public health approach: A scoping review and narrative synthesis
Source: PLOS Glob Public Health. 2024 Jan 10;4(1):e0002790. doi: 10.1371/journal.pgph.0002790 (PMC10781110; doi:10.1371/journal.pgph.0002790)
Supplement: S4 Table — (DOCX) [file pgph.0002790.s005.docx]

S4 Table: Publication details of those included studies that reported applying a public health approach to violence, including crime and delinquency, with details of how each has been operationlised (n=19)

| **Study** | **Application/ intervention name(s) with location** | **Aim of the intervention** | **Rationale for PH approach** | **Nature of the violence** | **Focus on Primary/secondary/tertiary prevention?** | **Anticipated impact?** | **Population targeted or universal?** | **Cross-sector working/ collaborations?** | **Evaluation done? If so, what results** | **How they have attempted to deliver at scale? Or recommendations made?** |
| --- | --- | --- | --- | --- | --- | --- | --- | --- | --- | --- |
| Linton et al. (2010) [1] | North Carolina Sexual Violence Prevention Plan and Rape Prevention and Education (RPE) program: includes Bystander Approach; in 3 regions of North Carolina, USA | To increase sustainable primary prevention programming;  To create buy-in among community members; | Scale of the problem and consequences for victims, families, friends and society; | Sexual violence | Primary - Bystander approach; Adaptations for education/curricula using RPE prevention coordinators; stronger state laws, policies and procedures | Neighbourhood level (by region/ statewide) | statewide universal programmes (Bystander approach and education/curricula adaptation in schools) | Yes, the North Carolina Division of Public Health’s Sexual Violence Prevention Team (NCSVPT) is an interdisciplinary group of stakeholders representing universities, domestic violence and rape crisis centers, community educators, the North Carolina Coalition Against Sexual Assault, and PH practitioners; | limited, uses the ‘most-promising programs’ (primary -Bystander Approach)  3 regions focus on different approaches with limited preliminary evaluation results | RPE programs reportedly implemented in 3 different regions: Recommendations include community-based task forces, assessing their communities’ needs and strengths, and implementing and evaluating strategies designed to change attitudes, behaviors, and community norms supportive of sexual violence. |
| Picard-Fritsche & Cerniglia (2013) [2]  (from review paper [3] ) | Crown Height’s Save Our Streets (SOS) including community mobilization campaign and street-level outreach, violence interruption activities (replicate of Chicago Ceasefire, CV model), in neighbourhoods in Brooklyn New York, USA | Widening decision alternatives and enhancing the perceived costs of risky behaviour for the for high-risk target groups; mobilize community leaders, clergy, residents, and law enforcement to change community-wide norms and perceptions related to gun violence. | Scale of the problem; Replication of Chicago Ceasefire project which significantly reduced incidences and density of gun violence in some other communities; | Gun violence | Primary – community-wide educational campaigns to modify community norms to gun violence;  Secondary – violence interrupters and street conflict mediation (includes Social determinates of Health, SDH, as outreach workers may link high-risk participants to social services: job training, employment assistance, education);  Tertiary – organising vigils within 72 hrs of a shooting as community mobilisation | Individual, family and neighbourhood | Targeted community and high-risk individuals | Yes, staff working in collaboration with local stakeholders and staff of the Chicago Project on Violence Prevention; | Yes, average monthly shooting rates in Crown Heights decreased by 6% from the pre to the post periods, while increasing in the three comparison areas between 18% and 28%; gun violence in Crown Heights was 20% lower than what it would have been had gun violence trends mirrored those of similar, adjacent precincts; process evaluation of impact: not a strong or statistically significant relationship between the program and the answers to questions about gun violence norms; | The authors report that given the close adherence of SOS to the original Chicago Ceasefire model, this finding supports the ongoing replication efforts currently in progress across New York City and the rest of the country. |
| Wagman et al. (2012) [4] | Safe Homes And  Respect for Everyone (SHARE) Project: adapted from Raising Voices and Stepping Stone (for IPV) prevention strategies in 4 regions of Rakai, Uganda | To transform community attitudes about gender norms and acceptability of partner abuse; raising awareness about women’s rights and the negative consequences of IPV on women, children, relationships, and communities | ‘IPV is common and *attitudes condoning* it are widespread’; numerous adverse consequences for victim; and others indirectly effected; adverse economic consequences | Intimate Partner Violence (IPV) | Primary – community activism course: advocacy, capacity building, learning materials and special events | Neighbourhood/ community level | Universal and targeted (Kojja Program for young men to raise awareness about non-violent approaches to problem solving and conflict resolution) | Establishment of Local network collaborations, community action groups;  Strategy-specific activities were tailored to match the communities’ readiness for behavior and attitude change at each stage of the process, as measured through systematic monitoring and evaluation done by SHARE staff, volunteers, and partners | used multiple Adapted Promising Practices (Raising Voices to developed a Resource Guide’s Community Activism Course and Stepping Stones which is a participatory training program). Study evaluated the implementation practicalities, but no outcome effectiveness results given. | Provide support for personal and professional development of all personnel from the start;  7 reported recommendations for wider roll out |
| May (2013) [5] | Preventive counselling for gun violence, USA | Alerting high-risk group of patients to situations that increase risk of gun injury/death | Physicians’ engagement in preventive health issues | Gun violence | Primary - routine clinical setting | Individual level | Targeted population only (age risk age group African American men aged 18-35 year old) | No | Very limited to post encounter interview | none reported |
| Webster et al. (2013) [6] | Baltimore Safe Streets program (replicate Chicago’s CeaseFire program given as an Evidence-Based PH program) in 4 neighbourhoods (police posts) in Baltimore, USA | To change behaviours, attitudes, and social norms, of youths, most directly related to gun violence in top quartile for number of homicides and non-fatal shootings during 3 yrs prior (ie. high-risk community) | Scale of the problem;  Replication of Chicago’s CeaseFire program; | Gun violence | Primary – street workers work with high-risk clients: connecting youths to educational and job opportunities (SDH); Community events  Secondary – street workers to mediate conflict; | Individual and neighbourhood level | Targets communities at high risk of gun violence (street outreach workers would mediate conflicts as well as work with high-risk youth clients) | Prior to program implementation, CeaseFire staff provided extensive training to Safe Streets program staff;  Collaboration with Baltimore City Health Department (BCHD), Baltimore Police Department, for outcome data, and community-based organisation | Yes, with primary outcomes homicides and nonfatal shootings; Although estimates of program effect varied, three of the four intervention neighborhoods experienced program-related reductions in at least one measure of gun violence without also having a statistically significant increase in another measure of gun violence. ~5.4 fewer homicide incidents, ~ 35 fewer nonfatal shootings and at least five homicides across 112 cumulative months of program implementation across the four sites. | None given as this was reported as a replicate of the Chicago’s CeaseFire program (minus specialised “violence interrupters”) |
| Erickson et al. (2014) [7] | The Wakanheza Project,  3 urban community organisations, USA | Promotion of healthier communities by addressing interpersonal dynamics of family violence using social strategies to change behavioural norms | Magnitude of violence impacting on families and children; PH needed to address this multisystemic problem; | Family violence | Primary – alteration of physical environment in public/community spaces | Family level | Targeted large public places at risk of family violence | Collaboration with staff of community organizations, agencies, that serve families, children, and youth | Some findings of immediate impact on physical environment and organisational culture as well as staff were reported and described; | Includes focus groups of staff members of agencies to understand their involvement in implementation: Resulted in descriptions for Initiating Events; Initial Implementation events; impacts/changes to physical environment and organizational culture, strategies and practices for sustaining the Wakanbeza Project, Individual impacts and changes, allowing possible replication |
| Williams et al. (2014) [8] | Community Initiative to Reduce Violence (CIRV): part of the Scottish Violence Reduction Unit (VRU), Glasgow, Scotland, UK | High-risk male individuals accessed services (SDH) and opportunities for compliance with ‘no violence, no weapon pledge’ with social relationships as positive force. | Extent of problem & long history of youth gangs; based on successful US Cincinnati initiative that used focused-deterrence approach that provided social support; | Gang-related violence | Primary  ** acts on SDHs such as employment, personal development and housing so SYSTEM APPROACH | Individual, family and neighbourhood | Targeted high risk offenders of physical violence and weapon carriage | Led by the Strathclyde Police with collaboration with existing statutory services (education, housing, social services and health) and third sector parties (that provide diversionary activities, personal development and job-readiness) after ‘needs analysis’ to provide client support. | Diversionary activities- first preliminary evaluation: In the study area, young men who engaged with CIRV greatly reduced their carriage of weapons (principally knives) was statistically significantly greater than that of the age/sex matched comparison group; gang-related youth reduce their rate of physical violence after engagement with CIRV | The authors reported that the intervention was tailored to the nature of the problem and the resources either already available or made available through specific funding (p690) |
| Freire et al. (2015) [9] | Domestic Violence Prevention Enhancements and Leadership  Through  Alliances (DELTA) Program, Preparing and Raising Expectations for Prevention (PREP), USA | To build on organisational prevention capacity; Interactive Systems Framework (ISF) posits that both general and innovation-specific capacities are necessary to successfully support and deliver prevention programs | History of extent of IVP; consequences of long-term health and social costs; framing IPV within a social ecology | Intimate partner violence (IPV) | Primary – aim to prevent first-time perpetration or victimisation (no mention of SDH use) | N/A (no individual prevention interventions reported) | Targeted and universal | Domestic violence coalitions from 19 states to build organizational prevention capacity and catalyze Intimate Partner Violence (IPV) primary prevention strategies within their states in partnership with external partners or local member agencies. | No effectiveness evaluation of individual programs or coalitions but evaluation using a measure 10-item index of Prevention Capacity of the organisation/ coalition (table 3); DELTA PREP achieved its capacity-building goal, with all 19 participant coalitions improving their prevention capacity. | The project provided eight support areas to facilitate coalitions’ developing and implementing action plans for organisational change (yr1-3) and prevention activities (yr2-3);  The project’s flexible approach was developed to support the ability to engage with coalitions at different prevention capacity levels and to find combinations of supports that could meet the needs of most coalitions. |
| Crosby and Lyons (2016) [10] | National Violent Death Reporting Tool by Centres for Disease Control and Prevention (CDC): homicides prevention from legal intervention in 17 states in USA | linking data from various state and local agencies and sources to be used in creation of new initiatives. | Differences in recording and surveillance tools between states | Violent death/ homicides | Primary - Survey results to understand the patterns of violence and the factors associated with its occurrence to strategically target at-risk populations | Neighbourhood/ state level | Universal (linking of multiple data sources) | State surveillance system that links data from various state and local agencies and sources: medical examiners or coroners, law-enforcement agencies, and death certificates.  Participating states have used the NVDRS data in partnership with prevention-program implementers to create new initiatives and adjust existing programs | Not evaluated as aimed to understand key risk factors and protective factors to why homicides occurs and whom it affects thus improve prevention strategies as based on data. | Not given although the authors reported that CDC is striving to make NVDRS data more accessible for analysis and use by researchers, prevention program implementers, and policy makers (p1511) |
| Duckworth (2016) [11] | Domestic Violence Prevention Enhancement and Leadership Through Alliances (DELTA) Focusing on Outcomes for Communities United with States (FOCUS) by CDC and the Delaware Coalition Against Domestic Violence (DCADV): 14 state, US | emphasis on implementation and evaluation to help build practice-based evidence; state-level strategies aimed at impacting system norms within the PH healthcare; to promote positive social norms; | History and unknown extent of “private acts of violence happening in homes and relationships” | Domestic violence/IPV | Primary – Project P.I.N. (Performing Informing Norming) & bystander intervention; community trauma-informed, supporting community healing, resilience and healthy resistance strategies; to improve **SDH** | Neighbour level | Universal approach:  Performing, Informing, Norming (P.I.N.) school and community-level strategies using bystander interventions; | Implementation of local-level strategies by third sector companies;  Creation of ‘Trauma Matters Delaware’ steering committee made up of state and community-based agencies;  Many partnerships and partners are listed from multi-sector agencies, in the DCADV capacity-building timeline; With difference strategies linked to different sectors: P.I.N. developed in partnership with Art Fusion Inc. | No, description of innovative, strength-based strategies and local-level strategies given but no evaluation results reported. | Some details as DCADV has developed webinars and trainings to support community and state partners in adopting this ‘Connecting the Dots’ framework that is increasingly being championed by funders and policy makers;  Safe + Respectful, a Delaware DELTA program implemented by Child, Inc., is selected as a CDC case study to be disseminated nationally |
| Tibbs et al. (2017) [12] | Safe Streets Baltimore initiative funded by Baltimore City Health Department (BCHD) Baltimore, USA | shift the discourse toward the view that violence is a disease by placing the emphasis on prevention and finding solutions to end the epidemic | Response to a 30-day period of high level of violence and civil unrest; | Youth violence | Primary – community events  Secondary – mediate and deescalate conflicts | Individual, Neighbour level | Targeted communities at high risk of violence occurring | BCHD coordinate and collaborate with community-based organisations and institutions including Associated Catholic Charities, Family Health Centers Baltimore, Living Classrooms, and Park Heights Renaissance which provide site management, hiring and overseeing staff and offering unique resources to supporting the community. | Preliminary data indicate that the BCHD has been able to implement an effective community-wide strategy to prevent violence, particularly among youth, with 55% percent of mediations occurred before the conflict escalated to violence (p642) | An aspect of the program was to identify any additional areas of need in the community with the programme being adaptive to meet those need ie. They hired a female outreach worker or implemented a Hospital-Based Violence Intervention project thus providing wraparound support to person’s affected by violence. |
| Cerda et al.  (2018) [13] | Save Our Streets (Cure Violence model in Crown Heights, Brooklyn) & ‘hot-spot’ policing in 14 neighbourhoods, New York City, US | (1) working with friends and families of victims to prevent retaliation and mediate ongoing disputes; (2) changing the thinking of those at highest risk of perpetrating violence; and (3) changing group norms about violence in the broader community | Scale of problem; mixed findings and limited evidence; used to estimate relative impact of CV and hot-spots policing | Urban/ youth violence | Primary – mentoring high-risk individuals and connecting them to job and educational opportunities (SDH)  Secondary – mediating and deescalating conflicts  Tertiary – stopping retaliations between residents or gangs | Individual, family, neighbourhood/ community level | Targeted communities at high risk of violence; changing group norms about violence in broader community | Collaboration across multiple sectors, with community-based organisations and outreach workers | Evaluated by using a simulation modelling technique: homicide could decrease by about 24% over 20 years with standard investment in Cure Violence in violent neighborhoods, or with more than doubling the police force over the same time period; Combined approach (CV and policing) was incorporated in the model and gave results that could achieve more, with fewer resources over a shorter period of time that either intervention alone. | the authors reported that the model was specific to New York City and hence generalizability to other contexts may be limited. |
| Chen (2018) [14] | Violence Prevention Alliance of the World Health Organisation in Wilmington, Delaware, US | To apply social ecological framework in approaching violence prevention that recognizes that prevention must address individual, relationship, community, and societal factors | Scale of problem (firearms injuries at all-time high in 2017); | Gun violence | Secondary – mediating and deescalating conflicts by violence interrupters  Tertiary – stopping retaliations between residents or gangs | Individual, family neighbourhood/ community levels | High risk community/ key offenders | Organisation partnership and Wilmington Community Advisory Council coalition of 38 representatives from public, private, government and non-profit agencies | No, one of the critical next steps will be designing a blend or “package” of well-planned programs (Focused Deterrence programs; Hospital Violence Intervention Programs (HVIPs), Cure Violence) and evaluations to address populations at different levels of risk in ways that are careful not to profile or stigmatize, especially those at the highest risk of injury. | Scalability of interventions was reportedly linked to the capacity of each community: High capacity communities that already had active intervention programs, ‘packaging’ meant aligning existing resources and adding complementary programs.  Low capacity communities, where either no interventions existed or could not be easily scaled, trust did not exist between potential partners, or took significant time to build capacity before program implementation could begin. |
| Harris (2018) [15] | Scottish’s VRU including Medics Against Violence in Scotland, UK | W | Individual case studies; scale of problem/ incidences/ outbreaks | Knife/ youth violence | Primary – targets those suspected of gang involvement with education and information campaigns & given a ‘way out’ through help with housing, relocation, employment and training (**SDH**)  Tertiary - support for perpetrators (Navigator) | Individual and family level | Universal and targeted | Yes, intelligence and data shared between police and others working in health, education, youth work.  Medics Against Violence charity affiliated to the VRU - Healthcare professional, including school nurses, to lead education and early interventions within schools to show the consequences of knife violence to pupils from different perspectives.  Navigator (hospital-based intervention programme in emergency departments) for 16plus patients whereby ‘Navigators’ help them see implications of knife carrying. | Yes : number of homicides in Scotland fell by 47% between 2007-08 and 2016-17, from 115 to 61, and no one under the age of 20 was killed with a sharp instrument in 2016-17 | Some, collection of easily accessible online resources/tools giving practical advice and range of approaches, with varying amounts of evidence of success, for schools and colleges about preventing youth violence and gang involvement; online strategy documents about Redthread and Navigator and associated programs and interventions; |
| Astrup (2019) [16] | VRUs in London and Wales | To understand and address the complex root causes behind violence, as well as responding to incidents of violence and working towards long-term rehabilitation | Scale of problem “knife crime epidemic”; in response to success of Scottish VRU;  extra funding from the serious violence strategy to support programmes and community partnerships | Knife/ youth violence | Primary – youth advocates programme; major media campaigns such as #knifefree  Secondary – ‘PH duty’ by professional to raise any concerns.  Tertiary – RedThread charity with youth workers in A& E units in London to intervene with young people following incidents of violent incidents | Individual level | Universal and targeted (#knifefree major media campaign targets teenagers to set out risks of carrying a knife based on real life stories) | Violence Reduction Partnership with Merseyside;  Redthread charity placing specialist youth workers in A&E units in London, Nottingham and Birmingham.  ‘PH duty’ ensuring professionals across health, education, police, social services, housing and the voluntary sector are held accountable  Wales: Early Action Together – multi-agency partnership between PH Wales, 4 police forces, police and crime commissioners, Barnardo’s and HM Prison and Probation Service that uses a PH approach to policing and criminal justice to deal with vulnerable people and the root causes of criminal behaviour. | No, but refers to pioneering VRU in Scotland: Homicides north of the border more than halved, from 137 in 2004-5 (Violence Reduction Unit, 2019) to 59 in 2017-18, the joint lowest level for a 12-month period since 1976 (Scottish Government, 2018). | None given. The authors noted that a long-term strategy with all departments and agencies working together with local communities, using evidence-based approaches was needed  Recommends strong policing and PH approach as both needed. |
| Iacobucci (2019) [17] | Violence Reduction Partnership (previously VRU) in Merseyside, UK | To tackle the underlying factors behind a recent increase in serious crime | Partnerships to pool combined intelligence and resources diagnose the problem, better understand it, and then develop and deliver effective plans to most needy | Violent crime/ violence | Primary - tackling societal problems, including poverty, mental ill health, education, issues of addiction and lack of opportunity (SDH) | Individual, family, and neighbourhood level | Universal (multimedia campaigns, community engagement) and targeted (Navigator intervention) | Partnership between clinicians, the police and local government lead by independent chairperson; | start-up PH approach; strategies aimed to be underpinned by data and subject to ongoing evaluation against a range of indicators: school attendance, appearance in criminal justice system, mental health outcomes; | No but highlights objectives and need for benchmark data for the 10-year strategy |
| Kolbe (2020) [18] | K-12 Schools Security – School Emergency Operations Plan, USA | Operations plans are prepared to prevent threats to PH and safety using the Whole School, Whole Community, Whole Child school health framework | Scale of problem via current school gun violence surveillance systems; | School gun violence | Primary, Secondary and Tertiary – will be implemented as part of operations plan | Individual, family, and neighbourhood level | Universal to school populations | Each surveillance system may not measure the same variables in the same ways in the same populations;  ‘calls on federal agencies to collaboratively: define short- and long-term outcomes; develop means to track and monitor progress; identify leadership roles and responsibilities; and determine how the collaborative mechanism will be funded and staffed’ | reported as inherently difficult to assess the effectiveness of the many school gun violence interventions;  Although surveillance systems and databases are listed that describe the effects of school gun violence and some describe the implementation of interventions (gun legislation, state policies, bills and resolutions, plan for shooting, crime victimization and security) | Scaling up for major national actions: governmental and nongovernmental surveillance systems development to monitor effects and implementation of monitored interventions to reduce such effects; |
| Clemmow et al. (2021) [19] | Fixated Threat Assessment Center (FTAC), UK | Uses a deductive approach to detect fixated individual profiles of cases from data | Studying threatening and other problematic behaviour may outline warning signs of potential attacks | Stalking/ violence against individuals/ public figures | Primary, secondary, tertiary – decisions about behaviour and diagnosed mental disorders of individuals identified subgroups of concerning behaviour profiles which lead to actions by FTAC staff to manage behaviour | Individual (deactivate some of the drivers of violence) | Targeted population of Pathologically-fixated individuals | Collaboration via case management system by FTAC staff members including police mental health nurses, forensic psychologist and psychiatrists | No, as exploratory study to identify five profiles of concerning behaviour and implementing evidence-based interventions with identified individuals may deactivate some of the drivers of violence. | Generalizability of results may be limited to UK but policy and practice implications have relevance internationally;  calls for analogous fields to adopt a similar, multiagency, intelligence-sharing, approach to combatting the full spectrum of grievance fuelled violence. |
| Mayfield (2022) [20] | Community Violence Prevention Plan, CVPP: (included CV violence interrupter model; SAFE Charlotte; Health Alliance for Violence Intervention (hospital-based)) in Mecklenburg County, North Carolina, USA | To evaluate collective impact efforts using data collaboration.  To provides equitable resources, build networks and change norms for disrupting violence;  To influence social and health inequities stemming from structural racism; | Scale of problem and long-term consequences for individuals and communities.  In response to community advocacy | Community violence | Primary and secondary | Individual | Universal | Youth Advocate Program (YAP) local non-profit agency implemented CV;  Violence Prevention Data Collaborative was formed to leverage cross-sector resources for data sharing, analysis, reporting and evaluation in support of activities aligned with the CVPP; wide range of 45 partners across local agencies, sectors, and programs to increase the community’s capacity for data-informed decision making and advance research that deepens understanding of complex community issues | CV application itself was not evaluated | The approach given was for setting up cross-sector data sharing, so the interventions can be evaluated. This was reported that this data sharing approach can be replicated and scaled to support cross-sector collaborations. |

Abbreviations: BCHD Baltimore City Health Department; CV Cure Violence; CVPP Community Violence Prevention Plan; DELTA Domestic Violence Prevention Enhancement and Leadership Through Alliances; FTAC Fixated Threat Assessment Center; IPV Intimate Partner Violence; ISF Interactive Systems Framework; NCSVPT North Carolina Division of Public Health’s Sexual Violence Prevention Team; SOS Save Our Streets program; PH Public Health; RPE Rape Prevention and Education program; SDH social determinants of health; SHARE Safe Homes And Respect for Everyone; UK United Kingdom; USA United States of America; VRP violence reduction partnership; VRU violence reduction unit; YAP Youth Advocate Program;

References

1. Linton B, Guerrero C, Przewoznik J. Primary prevention of sexual violence in North Carolina: a public health approach. North Carolina medical journal. 2010;71(6):557-8.

2. Picard-Fritsche S, Cerniglia L. Testing a public health approach to gun violence: An evaluation of Crown Heights Save Our Streets, a replication of the Cure Violence Model: Center for Court Innovation New York, NY; 2013.

3. Butts J, Roman C, Bostwick L, Porter J. Cure violence: a public health model to reduce gun violence. Annual review of public health. 2015;36:39-53.

4. Wagman J, Namatovu F, Nalugoda F, Kiwanuka D, Nakigozi G, Gray R, et al. A public health approach to intimate partner violence prevention in Uganda: the SHARE Project. Violence against women. 2012;18(12):1390-412. doi: 10.1177/1077801212474874

5. May J. A public health approach to gun violence. Annals of internal medicine. 2013;159(4):306.

6. Webster D, Whitehill J, Vernick J, Curriero F. Effects of Baltimore's Safe Streets Program on gun violence: a replication of Chicago's CeaseFire Program. Journal of urban health : bulletin of the New York Academy of Medicine. 2013;90(1):27-40.

7. Erickson C, Gault D, Simmons D. The Wakanheza Project: A Public Health Approach to Primary Prevention of Family Violence. Journal of Community Practice. 2014;22(1/2):67-81.

8. Williams D, Currie D, Donnelly P, Linden W. Addressing gang-related violence in Glasgow: A preliminary pragmatic quasi-experimental evaluation of the Community Initiative to Reduce Violence (CIRV). Aggression and Violent Behavior. 2014;19(6):686-91.

9. Freire K, Zakocs R, Le B, Hill J, Brown P, Wheaton J. Evaluation of DELTA PREP: A Project Aimed at Integrating Primary Prevention of Intimate Partner Violence Within State Domestic Violence Coalitions. Health education & behavior : the official publication of the Society for Public Health Education. 2015;42(4):436-48. doi: 10.1177/1090198115579413

10. Crosby A, Lyons B. Assessing Homicides by and of U.S. Law-Enforcement Officers. New England Journal of Medicine. 2016;375(16):1509-11.

11. Duckworth N. Paradigm Shift 2.0: A Coalition's Decade Long Journey Into The Public Health Approach to Violence. Delaware journal of public health. 2016;2(4):10-4.

12. Tibbs C, Layne D, Bryant B, Carr M, Ruhe M, Keitt S, et al. Youth Violence Prevention: Local Public Health Approach. Journal of Public Health Management & Practice. 2017;23(6):641-3.

13. Cerdá M, Tracy M, Keyes KM. Reducing Urban Violence: A Contrast of Public Health and Criminal Justice Approaches. Epidemiology (Cambridge, Mass). 2018;29(1):142-50.

14. Chen D. Firearm Violence in Wilmington. Delaware journal of public health. 2018;4(5):14-21.

15. Harris P. A FIGHTING CHANCE: THE JOURNAL OF THE HEALTH VISITORS' ASSOCIATION. Community Practitioner. 2018;91(7):36-41.

16. Astrup J. KNIFE CRIME:WHERE'S THE PUBLIC HEALTH APPROACH? Community Practitioner. 2019;92(6):14-7.

17. Iacobucci G. Merseyside launches public health approach to tackle rising violent crime. BMJ (Clinical research ed). 2019;365:l2253. doi: 10.1136/bmj.l2253

18. Kolbe L. School Gun Violence in the United States. The Journal of school health. 2020;90(3):245-53.

19. Clemmow C, Gill P, Corner E, et al. A data-driven classification of outcome behaviors in those who cause concern to British public figures. Psychology, Public Policy, and Law. 2021:No-Specified.

20. Mayfield C, Siegal R, Herring M, et al. A Replicable, Solution-Focused Approach to Cross-Sector Data Sharing for Evaluation of Community Violence Prevention Programming. Journal of Public Health Management & Practice. 2022;28:S43-S53.
